# Supplementary material for: Dual Effects of TARP γ-2 on Glutamate Efficacy Can Account for AMPA Receptor Autoinactivation
Source: Cell Rep. 2017 Aug 1;20(5):1123–35. doi: 10.1016/j.celrep.2017.07.014 (PMC5554777; doi:10.1016/j.celrep.2017.07.014)
Supplement: Document S1. Supplemental Experimental Procedures and Figures S1–S4 [file mmc1.pdf]

Cell Reports, Volume *20*

Supplemental Information

Dual Effects of TARP γ-2 on Glutamate Efficacy Can Account for AMPA Receptor Autoinactivation

Ian D. Coombs, David M. MacLean, Vasanthi Jayaraman, Mark Farrant, and Stuart G. Cull-Candy

FIGURE S1

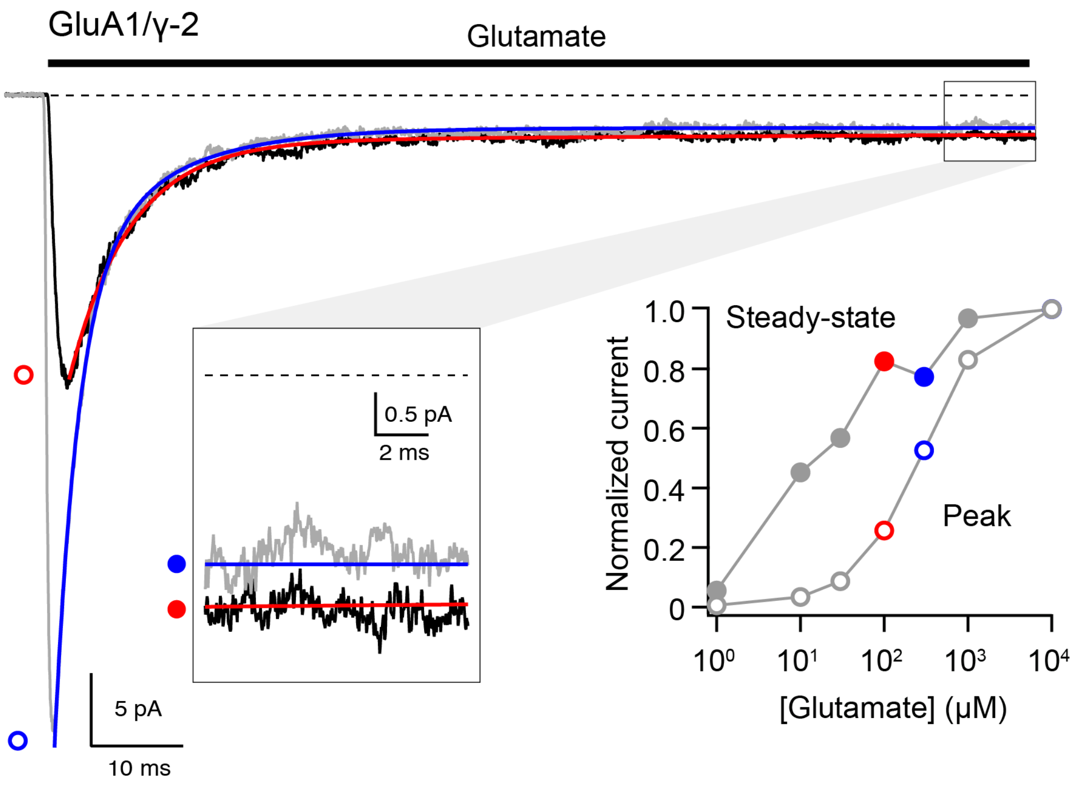

Figure S1 (related to Figure 1). Enhanced steady-state GluA1/γ-2 current at 100 versus 300 µM glutamate underlies the autoinactivation of the steady-state concentration response curve.
GluA1/γ-2 currents elicited by fast application of 100 µM glutamate (black record, red biexponential fit) and 300 µM glutamate (grey record, blue fit). Left-hand inset highlights the inverted concentration dependence of the steady-state current. Right-hand inset shows the contribution of these two records to both concentration-response relationships.

FIGURE S2

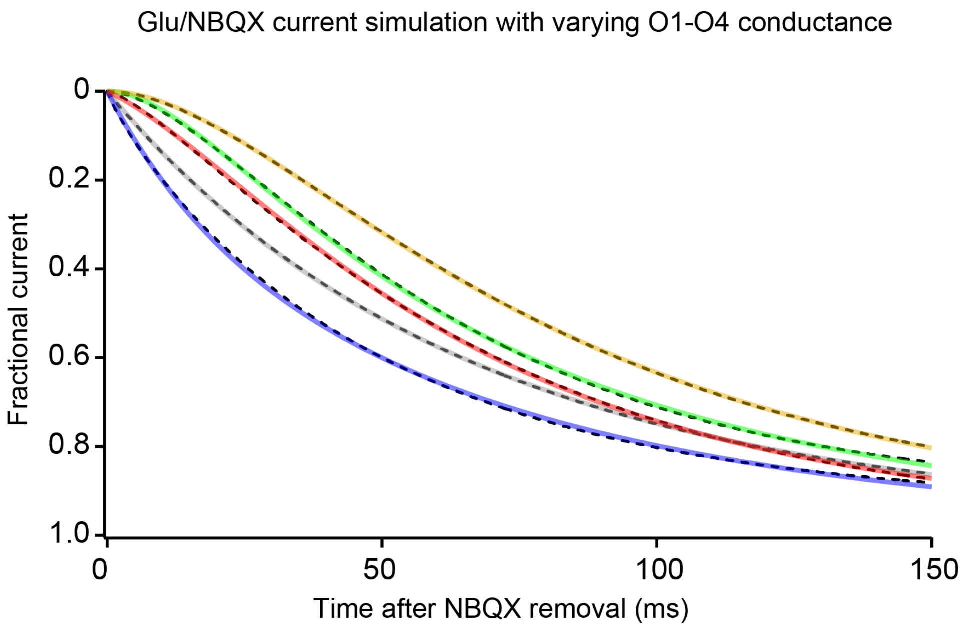

	Conductance (pS)					
	**O1**	**O2**	**O3**	**O4**	**'*m*'**	
	0	5	15	23	1.97	(Rosenmund et al.)
	0	9	15	21	1.77	(Robert and Howe)
	3.7	16.1	30.6	38.6	1.38	(Figure 5)
	5	10	15	20	1.00	
	10	15	20	25	0.87	

Figure S2 (related to Figure 5). The Hodgkin-Huxley exponent '*m*' of NBQX/glutamate currents is influenced by the relative subconductance levels of states O1–O4.
NBQX/glutamate currents simulated using the scheme in **Figure 5A** ($k_{NBQX} = 20\ s^{-1}$) demonstrating how the shape is influenced by subconductance levels of different relative magnitudes. Simulated curves were fitted (dashed lines) as in **Figure 5B**. Using measured subconductance levels for TARP-free AMPA/KARs (Rosenmund et al., 1998 - yellow) or those of Scheme 1 (Robert and Howe, 2003 - green) result in an *m* that approaches 2. Simulation using the sublevels from our single-channel recordings (red) yields a value of *m* (1.38) that is similar to that from our fits of TARPed macroscopic data (**Figure 5F**). When all states make a contribution proportional to their occupancy (grey) '*m*' is 1. If O1 makes a larger relative contribution (blue) '*m*' becomes less than 1 (for comparison, with GluA1/2(R)/γ-2, $m = 0.91 \pm 0.05$).

FIGURE S3

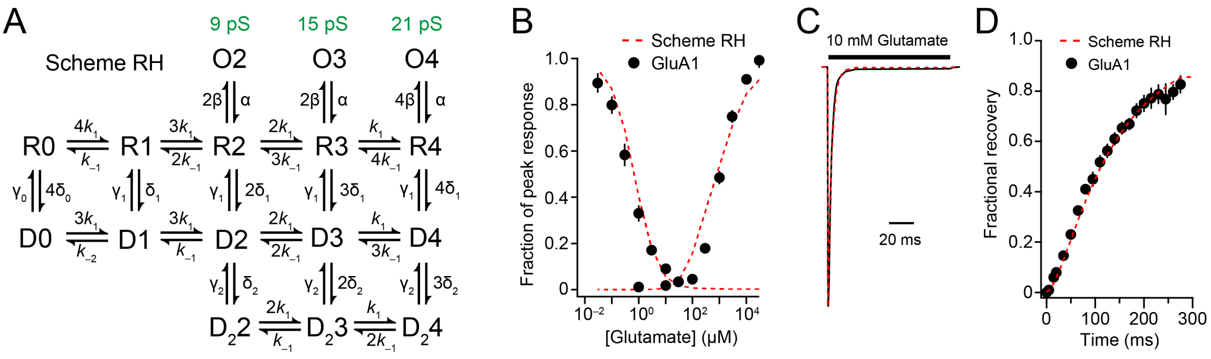

Figure S3 (related to Figure 6). A previously proposed model for GluA1 adequately describes our GluA1 data.

(**A**) Scheme RH, proposed by Robert and Howe (2003). All receptors (R0-R4) can desensitize (D0-D4). Receptors bound to two or more glutamate molecules (R2-R4) can additionally open (O) or enter a 'deep' desensitized state (D_2). With this scheme, a single set of rate constants can simultaneously approximate the two concentration response relationships (**B**), desensitization kinetics (**C**), and recovery kinetics of GluA1 (**D**) (red dashed lines). The following rate constants (constrained to be within 20% of previously published values) were used: k_1 = 1.6 x 10^7 $M^{-1}s^{-1}$; k_{-1} = 7400 s^{-1}; α = 2600 s^{-1}; β = 9600 s^{-1}; δ_1 = 1500 s^{-1}; γ_1 = 9.1 s^{-1}; δ_2 = 170 s^{-1}; γ_2 = 42 s^{-1}; δ_0 = 0.003 s^{-1}; γ_0 = 0.83 s^{-1}.

FIGURE S4

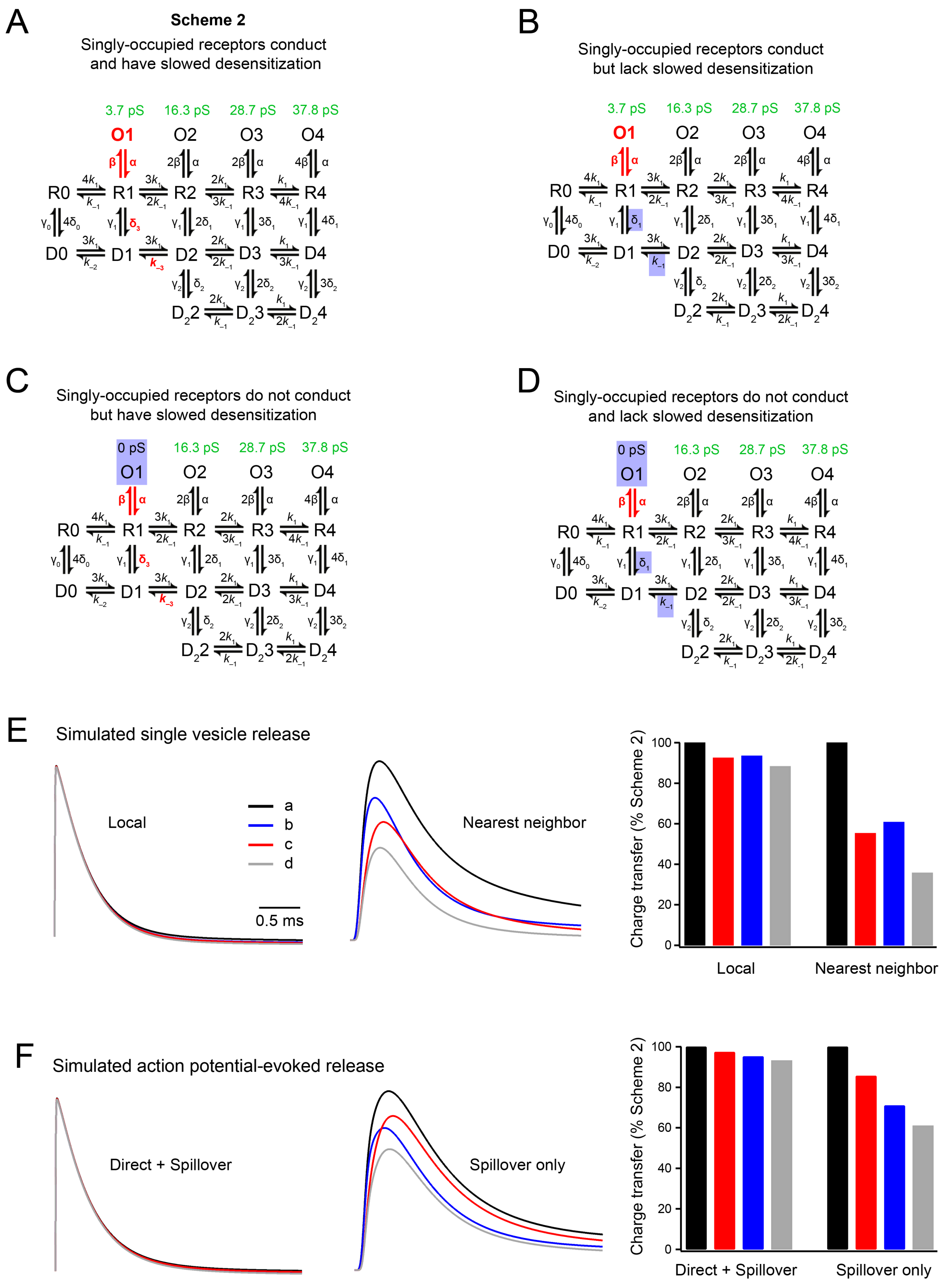

Figure S4 (related to Figure 7). Effects of γ-2 on both gating and desensitization of singly occupied receptors specifically increase charge transfer due to spillover.
(**A-D**) Kinetic schemes examining the relative contribution of changes to the conductance and kinetic behavior of singly occupied receptors (O1) to simulated synaptic charge transfer. (**A**) Scheme 2. (**B**) Scheme 2, altered to remove the slower desensitization of singly-liganded channels. (**C**) Scheme 2, altered to remove the

conductance of O1. (**D**) Scheme 2 with changes from A and B together. Blue boxes highlight the changed parameters. (**E**) Simulated single vesicle-induced currents resulting from kinetic schemes A-D (unboxed parameters as in **Figure 6**) using the glutamate waveforms shown in **Figure 7**. (**F**) As E, but for simulated action potential-evoked release. For both E and F, none of the scheme changes had marked effects on the charge transfer following local/direct activation (due to the high receptor occupancy in these conditions), but charge transfer of indirect activation was decreased by each of the individual changes and to a greater extent when both changes were made.

SUPPLEMENTAL EXPERIMENTAL PROCEDURES

Heterologous expression

We expressed recombinant AMPAR subunits and TARPs (plus EGFP) in HEK293 cells maintained under standard protocols, as described previously (Soto et al., 2007). AMPAR subunit cDNAs (rat) were 'flip' splice variants and the GluA2 forms were additionally R/G edited. Heteromeric AMPARs and AMPAR/TARP combinations were transfected at a cDNA ratio of 1:2 (GluA1:GluA2 and AMPAR subunit:TARP). The GluA1_γ-2 tandem consisted of full-length GluA1 and a 9 amino-acid linker (GGGGGEFAT) before the start codon of full-length γ-2. Transient transfection was performed using Lipofectamine 2000 (Life Technologies). Cells were split 12–24 h after transfection and plated on glass coverslips in the presence of 50 μM NBQX (Tocris-Abcam) to avoid AMPAR-mediated toxicity. Electrophysiological recordings were performed 18–24 h later.

Electrophysiology

Patch-clamp electrodes were pulled from borosilicate glass (1.5 mm o.d., 0.86 mm i.d.; Harvard Apparatus) and fire polished to a final resistance of 8–12 MΩ. For outside-out patches the 'external' solution contained 150 mM NaCl, 1 mM $CaCl_2$, 1 mM $MgCl_2$ and 10 mM HEPES, pH 7.4. The 'internal' solution contained 135 mM CsF, 33 mM CsOH, 11 mM EGTA, 10 mM HEPES, 2 mM $MgCl_2$ and 1 mM $CaCl_2$, pH 7.4 supplemented with 100 μM spermine tetrahydrochloride (Tocris Bioscience), except for NBQX/glutamate recordings where the internal solution contained 125 mM CsCl, 2.5 mM NaCl, 1 mM CsEGTA, 10 mM HEPES and 20 mM Na_2ATP. Recordings were made from outside-out patches at 22–25°C using an Axopatch 200A amplifier (Molecular Devices). Currents were recorded at –60 mV unless otherwise stated, low-pass filtered at 10 kHz and digitized at 20 kHz (Digidata 1440A interface with pClamp 10 software; Molecular Devices).

Rapid agonist application to excised patches

Rapid agonist application was achieved by switching between continuously flowing solutions, as described previously (Soto et al., 2014). Solution exchange was achieved by moving an application tool – made from either theta glass (2 mm outer diameter; Hilgenberg) or custom triple-barreled glass (Vitrocom) – mounted on a piezoelectric translator (Physik Instrumente) (MacLean et al., 2014; Robert and Howe, 2003). At the end of each experiment, the adequacy of the solution exchange was tested by destroying the patch and measuring the liquid-junction current at the open pipette (10–90% rise time typically 150–250 μs).

Data analysis

Concentration-response curves. Peak and steady-state concentration-response curves were constructed following application of multiple concentrations of glutamate to individual patches. Sensitivity to desensitization was derived from peak responses to 10 mM glutamate following equilibrium with various test concentrations of glutamate. Records were analyzed using Igor Pro 6.35 (Wavemetrics) with Neuromatic 2.8 (http://www.neuromatic.thinkrandom.com/), and curves from individual patches or pooled data were fit using the Hill equation to determine the concentration of glutamate generating the half-maximal response (EC_{50}) or half-maximal inhibition (IC_{50}). Each individual experiment was fitted separately and results were re-normalized to the peak of the fit. Pooled averages of the re-normalized results were refit for display only.

Conductance-voltage relationships. Responses to 2 s applications of 10 mM glutamate were obtained at various voltages between –110 mV and +60 mV. Peak and steady-state conductance-voltage (*G-V*) relationships were fitted with the Boltzmann equation:

$$G = G_{\text{max}} \left(\frac{1}{1 + \exp\left((V_{\text{m}} - V_{½})/k \right)} \right),$$

where G_{max} is the conductance at a sufficiently hyperpolarized potential to produce full relief of polyamine block, V_m is the membrane potential, $V_{½}$ is the potential at which 50% of block occurs, and k is a slope factor describing the voltage dependence of block (the membrane potential shift necessary to cause an e-fold change in conductance). Each individual experiment was fitted separately and data were re-normalized to the peak of the fit. Pooled averages of the re-normalized results were then refit for display only.

Fluctuation analysis. To deduce channel properties from macroscopic responses, glutamate (10 mM) was applied to outside-out patches (100 ms duration, 1 Hz). Non-stationary fluctuation analysis was performed as previously described (Soto et al., 2007) but using only the first 95% of the current decay. The ensemble variance of all successive pairs of current responses was calculated and the single-channel current (i) and total number of

channels (N) were then determined by plotting this ensemble variance (σ^2) against mean current ($\bar{I}$) and fitting with a parabolic function:

$$\sigma^2 = i\bar{I} - \bar{I}^2/N + \sigma_B^2 ,$$

where σ_B^2 is the background variance (Sigworth, 1980). To estimate the conductance of channels open at steady-state we performed stationary fluctuation analysis by determining the mean current and variance of the steady-state current. A line was fit between the origin (current = 0, variance = σ_B^2) and the single data point for the steady-state current, the slope of which provided an estimate of the single-channel current (i). In both cases, the weighted-mean single-channel conductance was calculated from the single-channel current and the holding potential.

Entry into, and recovery from, desensitization. For high concentrations of glutamate desensitization was determined directly from the fit of the current. For low concentration of glutamate, the rate of peak current inhibition was assessed using a two-pulse protocol jumping from control, into the test concentration for varying times and finally to 10 mM glutamate. Recovery from steady-state desensitization was measured by jumping from the test glutamate concentration to 10 mM glutamate following various intervals in control solution. Entry into desensitization was fitted with a sum of two exponentials for all conditions. Recovery from desensitization data was pooled, normalized and for GluA1 was fitted using a monoexponential Hodgkin-Huxley type equation:

$$I_t = (1 - e^{\left(\frac{-t}{\tau}\right)})^m ,$$

where I_t is the peak current at a given interpulse interval (t), t is the recovery time constant, and m is an exponent the value of which corresponds to the number of kinetically equivalent rate-limiting transitions that contribute to the recovery time course. Fits were standardized with a time constant of 120 ms to more clearly assess the delay of recovery (m). For GluA1/γ-2 recovery, we first established that a lag was absent (Hodgkin-Huxley m = 0.99) then performed a global biexponential fit, according to:

$$I_t = A_f \exp(t/\tau_f) + A_s \exp(t/\tau_s) ,$$

where A_f and t_f are the amplitude and time constant of the fast component of recovery and A_s and t_s are the amplitude and time constant of the slow component. The recovery endpoint was constrained to 1 and fast and slow time constants were common to all conditions. Due to the low levels of desensitization, data acquired during recovery from 1 µM glutamate were excluded from the global fitting and were fitted independently, using the time constants derived from fits of the other three concentrations. The resultant weighted time constants were calculated as follows:

$$\tau_w = \tau_f \left(\frac{A_f}{A_f + A_s}\right) + \tau_s \left(\frac{A_s}{A_f + A_s}\right)$$

AMPAR activation following unbinding of NBQX. NBQX/glutamate currents were recorded by fast application of 10 mM glutamate following full block by 50 µM NBQX, all in the presence of 50 µM cyclothiazide. Macroscopic currents were binned by amplitude, yielding 100 data points calculated from the average time of all points in each bin. This approach gave equal weighting to each phase of the current for fitting. Data were fitted with a monoexponential Hodgkin-Huxley type function. Records suitable for single-channel measurement were filtered at 500 Hz. Stable sweeps were identified and conductances were assigned an occupancy (O1–O4) based on their position in the step-wise sequence. Cumulative all point histograms (0.025 pA binwidth) for each distinguishable conductance level per sweep were created in Igor Pro. Following normalization, histograms for each conductance state were averaged.

Kinetic Modeling

Kinetic simulations were performed in Scilab 5.5.0. (Scilab Enterprises; http://www.scilab.org) using the Q-matrix method (Colquhoun and Hawkes, 1982). For each set of rate constants, currents were calculated from the occupancies of all conducting states at given time points multiplied by their unitary current. GluA1/γ-2 NBQX/glutamate currents were fitted using the measured conductances of O1–O4 and by varying k_{NBQX} (**Figure 5e**). Least squares improvement of approximation of three concentration-response relationships, desensitization across all concentrations and recovery kinetics at 10 mM and 10 µM was performed within Scheme 2 (**Figure 6**). Normalized, averaged GluA1/γ-2 desensitization data was parsed (to 35 datapoints) to make the necessary computation manageable. The weighting of poorly fitted components was increased manually to facilitate convergence in subsequent rounds. For Scheme 2, k_{-2} and k_{-3} were constrained by microscopic reversibility.

SUPPLEMENTAL REFERENCES

Colquhoun, D., and Hawkes, A.G. (1982). On the stochastic properties of bursts of single ion channel openings and of clusters of bursts. Philos Trans R Soc Lond B Biol Sci *300*, 1-59.

Sigworth, F.J. (1980). The variance of sodium current fluctuations at the node of Ranvier. J Physiol *307*, 97-129.
