# Supplementary material for: Dual Effects of TARP γ-2 on Glutamate Efficacy Can Account for AMPA Receptor Autoinactivation
Source: Cell Rep. 2017 Aug 1;20(5):1123–35. doi: 10.1016/j.celrep.2017.07.014 (PMC5554777; doi:10.1016/j.celrep.2017.07.014)
Supplement: Document S2. Article plus Supplemental Information [file mmc2.pdf]

Cell Reports

Article

Dual Effects of TARP γ-2 on Glutamate Efficacy Can Account for AMPA Receptor Autoinactivation

Graphical Abstract

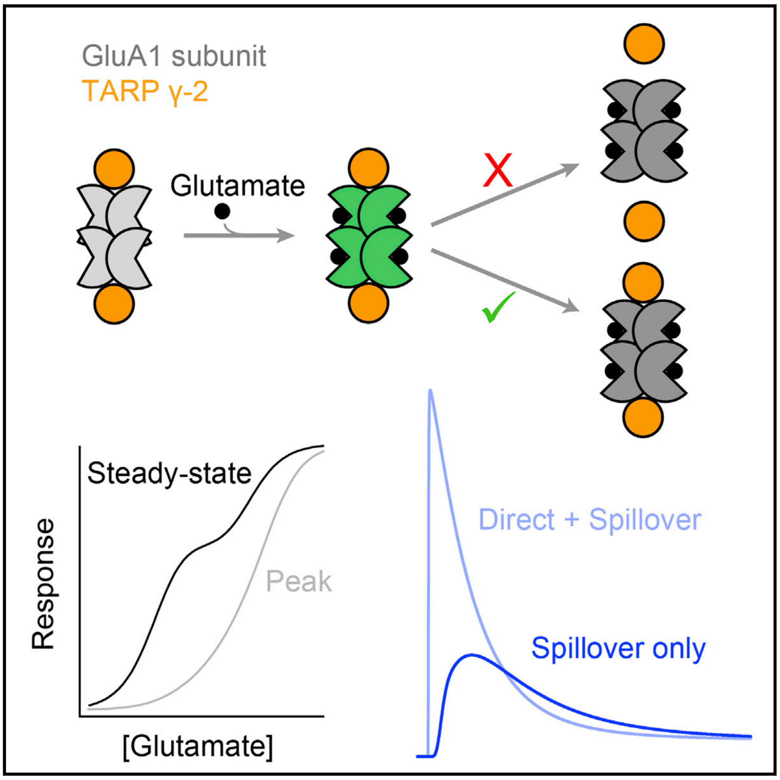

Highlights

- TARP γ-2 reduces desensitization and enhances the gating of singly liganded AMPARs
- This accounts for biphasic steady-state dose-response curves (autoinactivation)
- The effects of γ-2 are predicted to enhance synaptic spillover currents
- Desensitization does not lead to functional dissociation of the AMPAR/TARP complex

Authors

Ian D. Coombs, David M. MacLean, Vasanthi Jayaraman, Mark Farrant, Stuart G. Cull-Candy

Correspondence

m.farrant@ucl.ac.uk (M.F.),
s.cull-candy@ucl.ac.uk (S.G.C.-C.)

In Brief

AMPA receptors are regulated by accessory proteins, including TARP γ-2. Coombs et al. show how γ-2 can give rise to receptor behavior previously attributed to glutamate-induced dissociation of the AMPAR/TARP assembly. By favoring the gating of singly liganded receptors, γ-2 is predicted to facilitate synaptic signaling by low concentrations of glutamate.

Coombs et al., 2017, Cell Reports *20*, 1123–1135
August 1, 2017 © 2017 The Authors.
http://dx.doi.org/10.1016/j.celrep.2017.07.014

CellPress

OPEN ACCESS
CellPress

Dual Effects of TARP γ-2 on Glutamate Efficacy Can Account for AMPA Receptor Autoinactivation

Ian D. Coombs,[1,3] David M. MacLean,[2,3,4] Vasanthi Jayaraman,[2] Mark Farrant,[1,*] and Stuart G. Cull-Candy[1,5,*]
[1]Department of Neuroscience, Physiology and Pharmacology, University College London, Gower Street, London WC1E 6BT, UK
[2]Center for Membrane Biology, Department of Biochemistry and Molecular Biology, University of Texas Health Science Center at Houston, Houston, TX 77030, USA
[3]These authors contributed equally
[4]Present address: Department of Pharmacology and Physiology, University of Rochester Medical Center, Rochester, NY 14642, USA
[5]Lead Contact
*Correspondence: m.farrant@ucl.ac.uk (M.F.), s.cull-candy@ucl.ac.uk (S.G.C.-C.)
http://dx.doi.org/10.1016/j.celrep.2017.07.014

SUMMARY

Fast excitatory transmission in the CNS is mediated mainly by AMPA-type glutamate receptors (AMPARs) associated with transmembrane AMPAR regulatory proteins (TARPs). At the high glutamate concentrations typically seen during synaptic transmission, TARPs slow receptor desensitization and enhance mean channel conductance. However, their influence on channels gated by low glutamate concentrations, as encountered during delayed transmitter clearance or synaptic spillover, is poorly understood. We report here that TARP γ-2 reduces the ability of low glutamate concentrations to cause AMPAR desensitization and enhances channel gating at low glutamate occupancy. Simulations show that, by shifting the balance between AMPAR activation and desensitization, TARPs can markedly facilitate the transduction of spillover-mediated synaptic signaling. Furthermore, the dual effects of TARPs can account for biphasic steady-state glutamate concentration-response curves—a phenomenon termed "autoinactivation," previously thought to reflect desensitization-mediated AMPAR/TARP dissociation.

INTRODUCTION

Glutamate receptors of the AMPA subtype (AMPARs) mediate fast excitatory signaling throughout the mammalian brain (Traynelis et al., 2010). Typically, postsynaptic AMPARs are exposed to very brief glutamate transients that are thought to reach millimolar concentrations (Budisantoso et al., 2013; Clements et al., 1992), but at some synapses the intersynaptic diffusion of neurotransmitter results in receptors experiencing slower glutamate waveforms with peak concentrations in the micromolar range (Barbour and Häusser, 1997; Nielsen et al., 2004; Trussell et al., 1993). How the receptors respond to these different spatiotemporal glutamate concentration profiles shapes synaptic signaling (Jonas, 2000; Nielsen et al., 2004). The functional and pharmacological properties of AMPARs reflect the nature of their pore-forming subunits (GluA1–4) (Boulter et al., 1990; Geiger et al., 1995; Keinänen et al., 1990; Lomeli et al., 1994; Partin et al., 1996) and that of their associated auxiliary proteins, including transmembrane AMPAR regulatory proteins (TARPs) (Tomita et al., 2005), cornichons (Schwenk et al., 2009), cystine knot proteins (von Engelhardt et al., 2010), and GSG1L (Schwenk et al., 2012; Shanks et al., 2012). Notably, TARP γ-2 (stargazin) enhances agonist potency and efficacy, increases channel conductance, slows deactivation and desensitization, and reduces the voltage-dependent block of Ca^{2+}-permeable (GluA2-lacking) AMPARs by intracellular polyamines (Cho et al., 2007; Milstein et al., 2007; Priel et al., 2005; Soto et al., 2007; Tomita et al., 2005).

Each subunit of the AMPAR tetramer has a modular structure with an amino terminal domain, a ligand binding domain (LBD), a pore-forming transmembrane domain, and an intracellular C-terminal domain (Mayer, 2016; Sobolevsky et al., 2009). Assembled receptors interact with up to four TARPs (Hastie et al., 2013; Kim et al., 2010; Shi et al., 2009), primarily through transmembrane contacts running the length of the pore-forming regions as well as through contacts with the ligand binding domain (Shaikh et al., 2016; Twomey et al., 2016; Zhao et al., 2016). AMPAR interactions with the TARP intracellular C-terminal domain, its first extracellular loop (Ex1), and the transmembrane (TM) regions can all modulate multiple receptor properties (Ben-Yaacov et al., 2017; Cais et al., 2014; Dawe et al., 2016; Soto et al., 2014; Tomita et al., 2005; Turetsky et al., 2005). The clamshell-like LBDs are arranged as a dimer of dimers. Each LBD is able to bind a single glutamate molecule (Armstrong and Gouaux, 2000; Rosenmund et al., 1998), which stabilizes a more closed state of the clamshell (Landes et al., 2011; Ramaswamy et al., 2012; Zhang et al., 2008), producing tension in linkers connected to the pore (Kazi et al., 2014). When successive LBDs are closed, the pore generates subconductance levels of increasing amplitude, up to a main conductance when the receptor is fully liganded (Gebhardt and Cull-Candy, 2006; Rosenmund et al., 1998; Smith and Howe, 2000). In the continued presence of glutamate, the receptors desensitize because of rupture of the interface between LBD dimers, which relieves tension on the pore linkers and allows the channel to close (Armstrong et al., 2006; Meyerson et al., 2014; Sun et al., 2002). Desensitization can be triggered by just a single LBD closure (Robert and

This is an open access article under the CC BY license (http://creativecommons.org/licenses/by/4.0/).

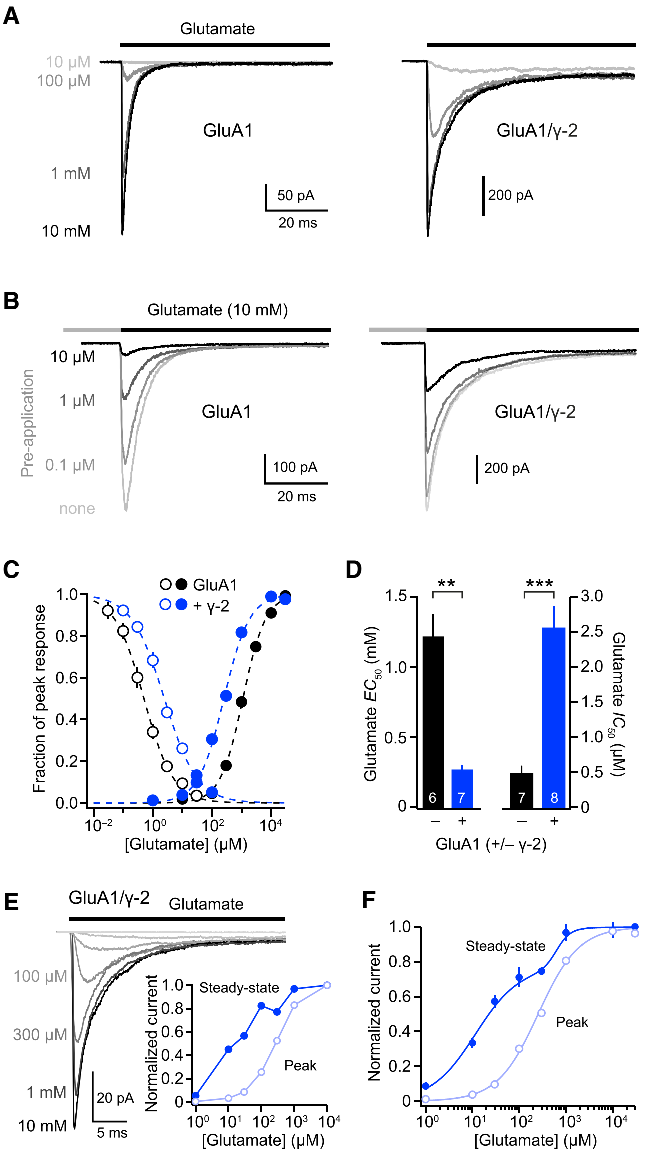

Figure 1. γ-2 Has Opposite Effects on Glutamate Potency for Activation and Desensitization

(A) Representative currents (–60 mV) elicited by fast applications of glutamate to outside-out patches from HEK293 cells transfected with GluA1 (left) or GluA1/γ-2 (right).

(B) Currents elicited by 10 mM glutamate following pre-desensitization by a range of lower glutamate concentrations.

(C) Pooled data fit with the Hill equation showing opposite γ-2-induced shifts in GluA1 potency for activation (filled circles) and desensitization (open circles). Symbols indicate mean and error bars indicate SEM.

(D) Pooled data showing glutamate EC_{50} for peak currents and glutamate IC_{50} for pre-exposure. Bars show mean and error bars show SEM.

(E) Representative GluA1/γ-2 currents. Inset: peak and steady-state concentration-response curves from the illustrated records. Note the inflection in the steady-state curve.

(F) Pooled data (n = 6 patches). Peak currents were well described by a single Hill function, and steady-state currents were best described by a double Hill function. Error bars denote SEM. $^{**}p < 0.01$, $^{***}p < 0.001$ (Welch t test).

See also Figure S1.

Howe, 2003). Interaction between the AMPAR LBD and the Ex1 loop of the TARP is thought to stabilize the channel open state and, thus, slow desensitization (Ben-Yaacov et al., 2017; Dawe et al., 2016; MacLean et al., 2014; Twomey et al., 2016; Zhao et al., 2016).

Despite the multiple regions of contact between AMPAR and TARP, it has been suggested that desensitization leads to a "functional uncoupling" of the TARP (Morimoto-Tomita et al., 2009; Semenov et al., 2012) or even the complete dissociation of TARP and AMPAR (Morimoto-Tomita et al., 2009). This proposal arose initially from the observation that the steady-state concentration-response relationship for GluA1 expressed with TARP γ-2 was biphasic, with a decline in current at high glutamate concentrations, a phenomenon termed "autoinactivation" (Morimoto-Tomita et al., 2009; Semenov et al., 2012). The physical separation of AMPARs and TARPs has been questioned (Semenov et al., 2012; Shaikh et al., 2016; Tomita, 2010). However, it has been proposed that desensitization-induced dissociation of AMPARs from synaptically anchored TARPs can modulate short-term synaptic plasticity by allowing the liberated receptors to diffuse from the post-synaptic domain and, thus, be rapidly replaced (Constals et al., 2015; Henley and Wilkinson, 2016; Morimoto-Tomita et al., 2009). Such a mechanism would have important implications for high-frequency central transmission, given the importance of AMPAR lateral mobility in maintaining the fidelity of the synaptic response (Heine et al., 2008).

Here we present an alternative mechanism that can account for autoinactivation without requiring changes in AMPAR-TARP interactions. We examined the effect of TARP γ-2 on the occupancy dependence of AMPAR gating and the concentration dependence of AMPAR desensitization. We show that γ-2 enhances the efficacy of glutamate by facilitating the opening of singly occupied receptors. Furthermore, we find that γ-2 reduces the sensitivity of GluA1 to desensitization by low concentrations of glutamate, slowing desensitization across all concentrations of glutamate. A kinetic model incorporating these dual effects of TARPs on glutamate efficacy that fully replicates our data suggests that TARPs amplify the transduction of spillover-mediated synaptic signaling and offers an alternative explanation for AMPAR autoinactivation with no requirement for desensitization-induced physical or functional uncoupling of the auxiliary subunits.

RESULTS

TARP γ-2 Reduces Desensitization of AMPARs by Low Concentrations of Glutamate

TARPs increase the potency of glutamate to activate AMPARs (Suzuki et al., 2008; Tomita et al., 2005), but their effects on the potency of glutamate to promote AMPAR desensitization have not been established. To address this, we recorded currents evoked by fast application of glutamate to outside-out patches from HEK293 cells transfected with GluA1 alone or with GluA1 plus γ-2 (GluA1/γ-2). From measuring peak current amplitudes we found, as expected, that γ-2 enhanced glutamate potency (Figures 1A and 1C). By contrast, there was a marked decrease in the ability of a pre-applied low concentration of glutamate to induce AMPAR desensitization ("pre-desensitization") and reduce peak currents (Figures 1B and 1C). Thus, although γ-2

produced an ~4-fold decrease in the glutamate concentration required for half-maximal peak current ($EC_{50,\ Pk}$) (from 1.2 ± 0.2 to 0.27 ± 0.03 mM, n = 6 and 7, p = 0.0017), the concentration of pre-applied glutamate required for half-maximal inhibition (IC_{50}) was increased 5-fold (from 0.48 ± 0.1 to 2.6 ± 0.3 μM, n = 7 and 8, p = 0.00019) (Figure 1D).

Steady-State Concentration-Response Relationships

Unlike cells transfected with GluA1 alone, where steady-state currents were too small to analyze, those transfected with GluA1 and γ-2 exhibited appreciable steady-state currents (Figure 1E). In all patches examined, the steady-state concentration-response relationships were biphasic, whereas the corresponding peak current relationships were sigmoidal (Figure 1F). Specifically, the steady-state relationships demonstrated a clear inflection at intermediate concentrations of glutamate, either at 100 μM (two of six patches) or 300 μM (four of six patches), producing a "shoulder" in the pooled concentration-response curve (Figure 1F; Figure S1).

Biphasic steady-state concentration-response curves, including bell-shaped curves with a clear peak at submaximal concentrations of glutamate, followed by a progressive decline at higher concentrations, have been reported previously for both native (Raman and Trussell, 1992) and TARPed recombinant AMPARs (Morimoto-Tomita et al., 2009; Semenov et al., 2012). This behavior has been termed autoinactivation and ascribed to a functional uncoupling of the AMPAR/TARP complex following desensitization-induced partial or complete dissociation of TARPs from AMPARs, although this interpretation remains controversial (Morimoto-Tomita et al., 2009; Semenov et al., 2012). The biphasic steady-state concentration-response relationship we measured could conceivably be interpreted as reflecting the presence of a mixture of TARPed and TARPless receptors rather than autoinactivation. However, this is unlikely because the peak concentration-response curve showed no evidence of a similar biphasic relationship.

Evidence for Maintained AMPAR/TARP Association

If desensitization were to induce functional uncoupling of the AMPAR/TARP complex, one might predict that this would lead to changes in multiple TARP-dependent AMPAR properties. To test this, we examined two such properties for both peak and steady-state currents—the voltage-dependent block by intracellular spermine (Soto et al., 2007) and the mean channel conductance (Soto et al., 2009; Tomita et al., 2005; Experimental Procedures). At steady state, both polyamine block (as judged by voltage of half-maximal block) and channel conductance (estimated from fluctuation analysis) were comparable with values obtained at peak (Figures 2A–2F). This result suggests that a majority of the steady-state current is mediated by AMPARs that remain functionally coupled to TARPs. However, although these experiments found no evidence for functional uncoupling, they do not refute its existence. The higher steady-state open probability of TARPed AMPARs would mean that they could contribute a majority of the equilibrium current even when they represented a minority of the receptor population.

We next searched for evidence of functional uncoupling under non-steady-state conditions using a different marker of TARP association—the enhanced efficacy of kainate at TARP-associated AMPARs (Tomita et al., 2005). It has been proposed that, following desensitization-induced functional uncoupling, the recovery of receptors from desensitization precedes TARP re-association (Morimoto-Tomita et al., 2009). If this is indeed the case, then the glutamate-evoked peak current (a measure of all non-desensitized receptors) should recover from desensitization more quickly than the kainate-evoked current (an indicator of TARP-associated non-desensitized receptors). However, following desensitization of GluA1/γ-2 by 10 mM glutamate, the recovery of both glutamate- and kainate-evoked currents displayed broadly similar kinetics (time constant of recovery of glutamate-evoked currents [τ_{Glu}] = 150 ± 20 ms, time constant of recovery of kainate-evoked currents [τ_{KA}] = 150 ± 10 ms, n = 5 and 4, p = 0.99; Figures 2G–2I).

Alternative Origins of Autoinactivation

In the absence of firm evidence to support functional uncoupling of TARPs from AMPARs, we next asked how else TARP-coupled receptors could generate biphasic steady-state concentration-response curves. AMPAR desensitization is known to result from rupture of the interface between LBD dimers following agonist binding (Armstrong et al., 2006; Meyerson et al., 2014; Sun et al., 2002). Indeed, in the absence of TARPs, there is compelling evidence that this can be triggered by the glutamate occupation of a single LBD (Robert and Howe, 2003). It has been proposed that TARPs stabilize LBD dimers, slowing desensitization (Priel et al., 2005), possibly mediated by interactions between the lower lobe of the LBD and the first extracellular loop of the TARP (Cais et al., 2014; Dawe et al., 2016; MacLean, 2013, 2014; Shaikh et al., 2016; Twomey et al., 2016; Zhao et al., 2016). We speculated that such γ-2-mediated stabilization might prevent efficient initiation of desensitization when only a single LBD is bound by glutamate, thereby enhancing occupancy dependence of the desensitization rate. Indeed, the decreased ability of pre-applied (low-concentration) glutamate to induce pre-desensitization of GluA1/γ-2 might be anticipated if desensitization became a co-operative process in the presence of γ-2, with singly occupied dimers desensitizing much more slowly than those that are doubly occupied.

We modeled this principle using a simple kinetic scheme with two agonist-dependent open, closed, and desensitized states (Figure 3A, scheme 1). Using rate constants from a previously proposed model of GluA1 (Robert and Howe, 2003), both peak and steady-state concentration-response curves were sigmoidal (Figure 3B). However, when we restricted the desensitization of singly occupied receptors, either by reducing the desensitization rates or increasing recovery rates, the steady-state concentration-response became bell-shaped (Figure 3C).

γ-2 Influences the Concentration Dependence of Desensitization and Recovery

To better understand the influence of TARPs on AMPAR desensitization and to determine whether the receptors did indeed display concentration-dependent properties that could account for autoinactivation, we next examined the effect of γ-2 on the onset of GluA1 desensitization over a range of glutamate concentrations. For concentrations of glutamate ≥10 μM, we

OPEN ACCESS
CellPress

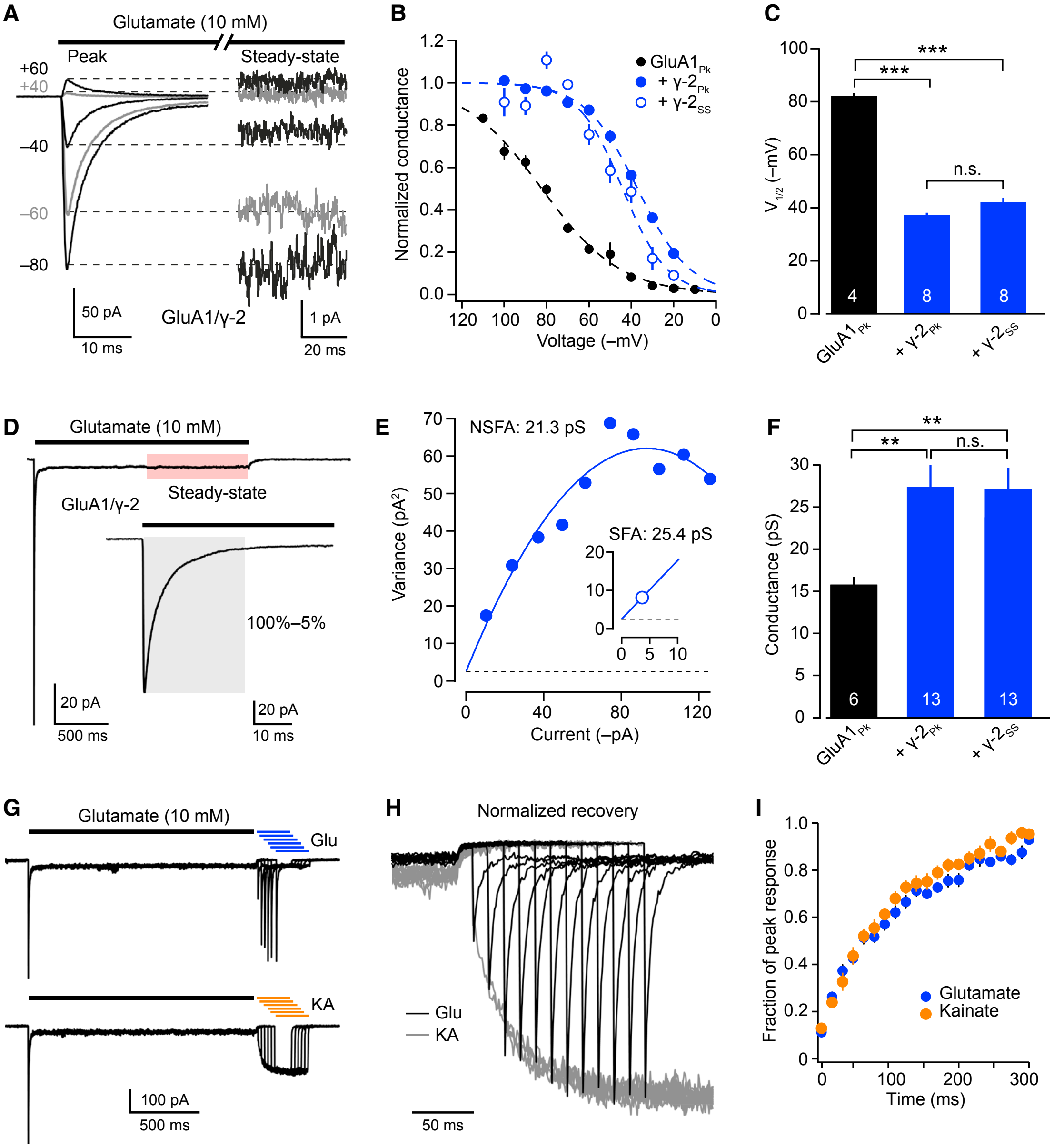

Figure 2. Measures of Conductance, Polyamine Block, and Kainate Efficacy Suggest Maintained AMPAR/TARP Association following Desensitization

(A) Representative GluA1/γ-2 *I-V* data. Peak and steady-state currents are scaled between 0 and –60mV.

(B) Conductance-voltage (*G-V*) plots of peak (GluA1 with- and without γ-2) and steady-state responses (GluA1/γ-2 only). Symbols indicate mean and error bars indicate SEM. Dashed lines are fits of the Boltzmann equation (Supplemental Experimental Procedures), from which values of voltage of half-maximal block ($V_{1/2}$) were determined. The steady-state current of GluA1 alone was too small to analyze.

(C) Pooled data showing $V_{1/2}$ of spermine block (one-way ANOVA, $F_{2,\ 8.7} = 311.53$, p < 0.0001). Bars indicate mean and error bars indicate SEM.

(D) Representative averaged glutamate-activated GluA1/γ-2 current (82 applications). Non-stationary fluctuation analysis (NSFA) was applied to the first 95% of the decay (inset, gray shading) and compared with stationary fluctuation analysis (SFA) of steady-state currents (red shading).

(E) Representative current variance plots for the decaying component (NSFA, filled symbols) and steady-state component (SFA, open symbol), yielding the indicated channel conductance estimates.

(F) Pooled data showing weighted mean channel conductance (one-way ANOVA, $F_{2,\ 18.9} = 14.51$, p = 0.00015). Bars indicate mean and error bars indicate SEM.

(G and H) Representative traces (G) and scaled responses (H) showing 10 mM glutamate- and 1 mM kainate-evoked currents as GluA1/γ-2 recovers from desensitization induced by 10 mM glutamate.

(I) Pooled data from four to five patches showing that the time course of recovery is the same for both agonists. **p < 0.01, ***p < 0.001 (Welch t test).

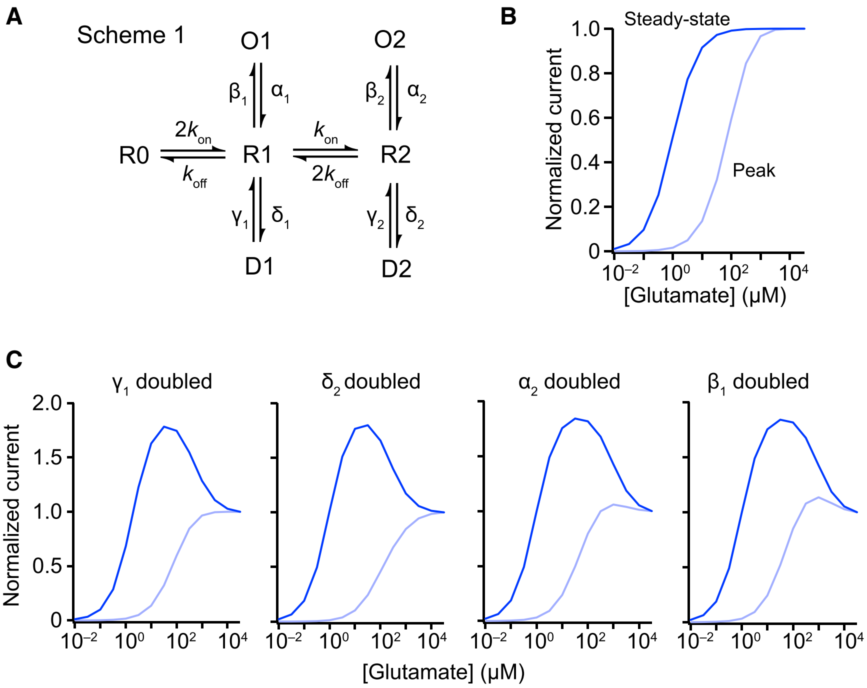

Figure 3. A Basic Kinetic Scheme Can Predict Biphasic Steady-State Concentration-Response Relationships

(A) A simple model, scheme 1, with two agonist binding steps (R0 → R1→ R2), two desensitized states (D1 and D2), and two open states (O1 and O2) with an equal conductance.

(B) Using rates based on previously published values for GluA1 (Robert and Howe, 2003; k_{on} = 2 × 10^7 $M^{-1}s^{-1}$, k_{off} = 9,000 s^{-1}, $\alpha_1 = \alpha_2$ = 3,100 s^{-1}, $\beta_1 = \beta_2$ = 8,000 s^{-1}, $\delta_1 = \delta_2$ = 1,800 s^{-1}, $\gamma_1 = \gamma_2$ = 35 s^{-1}), scheme 1 generates sigmoidal concentration response curves for both peak and steady-state activation.

(C) Decreasing occupancy of D1 relative to D2 (for example, increasing γ_1 or δ_2) or increasing the occupancy of O1 relative to O2 (for example, increasing α_2 or β_1) leads to biphasic steady-state concentration-response curves with minimal effects on peak concentration-response curves.

determined the kinetics of desensitization by directly fitting current decays (Figure 4A), whereas, for concentrations <10 μM, we measured the time course of peak current inhibition following glutamate pre-incubation (Figure 4B). The rate of onset of desensitization appeared to be independent of glutamate concentration above 300 μM, both for receptors with and without γ-2. However, it slowed markedly at concentrations below 100 μM (Figure 4C), and, unlike GluA1, GluA1/γ-2 desensitization was barely detectable at glutamate concentrations below 1 μM.

We also examined the effect of γ-2 on recovery from desensitization (Figures 4D–4F). The recovery of TARPless AMPARs from desensitization has been shown previously to exhibit a delay that can be fitted using Hodgkin-Huxley kinetics, where an exponent "*m*" > 1 indicates the occurrence of multiple, concurrent, kinetically similar rate-limiting steps (Robert and Howe, 2003). Consistent with this, our mean GluA1 recovery time course could be described by a monoexponential Hodgkin-Huxley (H-H) fit (Figure 4E). In the presence of γ-2, the recovery did not show a lag, and data were fitted with a simple double exponential (Figure 4F). Thus, for receptors containing γ-2, recovery from desensitization does not involve the same rate-limiting steps seen with receptors that lack γ-2. Of note, we found very limited concentration dependence of the recovery from desensitization (Figure 4F, inset). Taken together, these data show that GluA1/γ-2 displays a marked concentration dependence of entry into, but not recovery from, desensitization. As shown in Figure 2, such a decreased desensitization rate at low receptor occupancy is predicted to result in autoinactivation.

Glutamate Efficacy at Low Occupancy Is Increased by γ-2

To further assess glutamate efficacy at partially occupied receptors, we recorded glutamate-activated currents following pre-incubation with a competitive antagonist (Clements et al., 1998; Rosenmund et al., 1998). Although the binding of glutamate and the gating of AMPARs is fast (current rise times, ~200 μs), the unbinding of competitive antagonists such as 2,3-dioxo-6-nitro-1,2,3,4-tetrahydrobenzo[f]quinoxaline-7-sulfonamide (NBQX) is several orders of magnitude slower (MacLean et al., 2014). Thus, by saturating receptors with NBQX before fast application of glutamate, the time course and process of channel activation can be directly observed as NBQX molecules slowly unbind and are replaced by glutamate over a period of hundreds of milliseconds (Figure 5A).

Patches from cells expressing GluA1 or GluA1/γ-2 were initially exposed to 50 μM NBQX, followed by a rapid switch to 10 mM glutamate, all in the presence of cyclothiazide to prevent desensitization. We found that this "NBQX/glutamate" protocol resulted in macroscopic currents that displayed sigmoidal kinetics (Figure 5B), as previously reported for AMPAR-mediated currents evoked in patches from cultured hippocampal neurons in response to an analogous CNQX/kainate protocol (Clements et al., 1998). The observed delay in current onset is consistent with multiple agonist binding events being necessary to activate AMPARs (Robert and Howe, 2003; Rosenmund et al., 1998). To describe the rising phase of the responses, we fitted the currents with a mono-exponential Hodgkin-Huxley function (Experimental Procedures). For GluA1 alone, the currents could be described with a Hodgkin-Huxley exponent of 2.4 ± 0.1 (n = 6) (Figures 5B and 5C). This value suggests that more than two sequential binding events are necessary for full channel opening. On co-expression of γ-2, the required exponent was reduced to 1.4 ± 0.05 (n = 6, $p < 0.0001$), suggesting that fewer agonist binding events are necessary to gate AMPARs in the presence of TARPs (Figure 5C). A similar effect of γ-2 was seen when co-expressed with GluA2(Q) homomers or GluA1/2(R) heteromers (Figure 5C) and with the tandem construct GluA1_γ-2 (data not shown). Thus, association with γ-2 not only reduces

OPEN ACCESS
CellPress

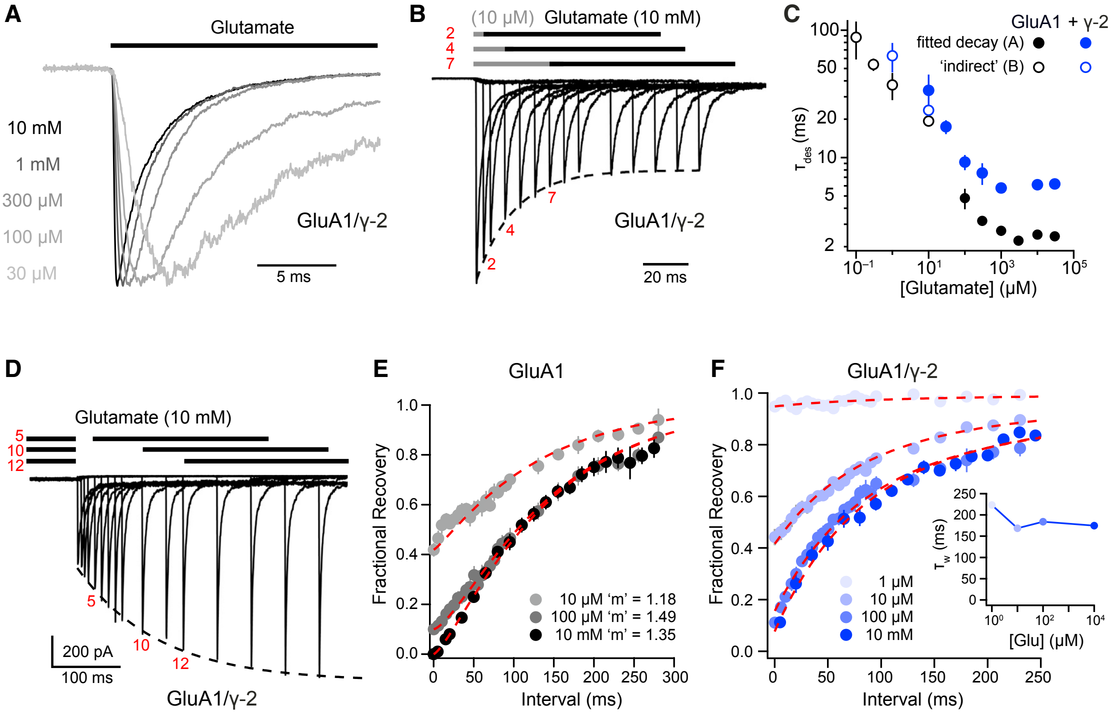

Figure 4. Concentration Dependence of GluA1/γ-2 Entry into and Exit from Desensitized States

(A) Representative normalized GluA1/γ-2 currents (–60 mV) showing markedly slowed desensitization at low glutamate concentrations (time constant of desensitization [τ_{des}] is 2.8 ms with 10 mM glutamate and 8.9 ms with 30 μM).

(B) Representative records showing the time course of entry into desensitization (jumping between control, 10 μM glutamate, and 10 mM glutamate). Three selected pulse protocols are illustrated. The dashed line is an exponential fit, giving a time constant of 26.6 ms.

(C) GluA1 (blue) and GluA1/γ-2 (black) desensitization kinetics measured using protocols of the type shown in (A) or (B) (n = 5–8; filled or open symbols, respectively). Symbols indicate mean and error bars indicate SEM.

(D) Recovery from desensitization by 10 μM glutamate measured using a two-pulse protocol. Three selected pulse protocols are illustrated. The dashed line is an exponential fit, giving a time constant of 119 ms.

(E) Monoexponential Hodgkin-Huxley fits of pooled averaged time courses of GluA1 recovery from desensitization by 10 μM, 100 μM, or 10 mM glutamate (n = 7). Recovery profiles are fitted with a single time constant (120 ms) with a variable *m* (Experimental Procedures).

(F) GluA1/γ-2 recovery from desensitization by a range of glutamate concentrations. Data were globally fitted with a double exponential function, giving fast time constant of recovery (τ_f) = 74 ms and slow time constant of recovery (τ_s) = 390 ms. Inset: the kinetics of recovery from desensitization; the weighted time constant of recovery (τ_w) is not markedly concentration-dependent.

desensitization but also substantially lowers the barrier to channel opening, resulting in enhanced gating of partially occupied AMPARs.

In four patches from cells expressing GluA1_γ-2 or GluA2(Q)/γ-2, where only a single channel was active and the background noise was sufficiently low, we were able to analyze in detail the subconductance levels. This allowed us to assign conductance values to the different states of occupancy (Figure 5D). Unlike TARPless receptors, which show three conductance steps in response to fast glutamate application following pre-incubation with NBQX (Rosenmund et al., 1998), with the TARPed receptors, we could resolve up to four sequential openings of increasing conductance (O1, O2, O3, and O4). Based on their position within the "staircase-like" sequence, we identified O4 in 100% of 134 sweeps, O3 in 93%, O2 in 83%, and O1 in 42%. That we were unable to identify O1 in all sweeps is to be expected, given that O1 has the lowest conductance, is the shortest-lived state, and can be identified unambiguously only when all three other states are resolved. The final weighted all-point amplitude histograms (Experimental Procedures) yielded conductances for O1–O4 of 3.7, 16.1, 30.6, and 38.6 picosiemens (pS) (Figure 5E). Incorporating these four conductance values into the NBQX-unbinding scheme (Figure 5A) provided an excellent fit to the macroscopic NBQX/glutamate responses (Figure 5F). Of note, for macroscopic currents from GluA1/2(R)/γ-2 heteromers, the Hodgkin-Huxley exponent (*m*) was less than that of homomeric receptors (Figure 5C). We performed simulations (Figure S2) that revealed that a reduced value of *m* can be indicative of an increased relative conductance of state O1. Thus, for the four states of GluA1/γ-2 (3.7, 16.1, 30.6, and 38.6 pS), the simulation yields an *m* of 1.38. However, if each state displays a conductance proportional to its occupancy (for example 5, 10, 15, and 20 pS), then *m* is precisely 1. Further, if the relative contribution of O1 is increased, then *m* can even be less than 1 (Figure S2). Although directly discerning modest differences in single-channel conductance states is not technically feasible for GluA1/2(R) heteromers (because of their low conductance), our macroscopic data suggest that O1 may

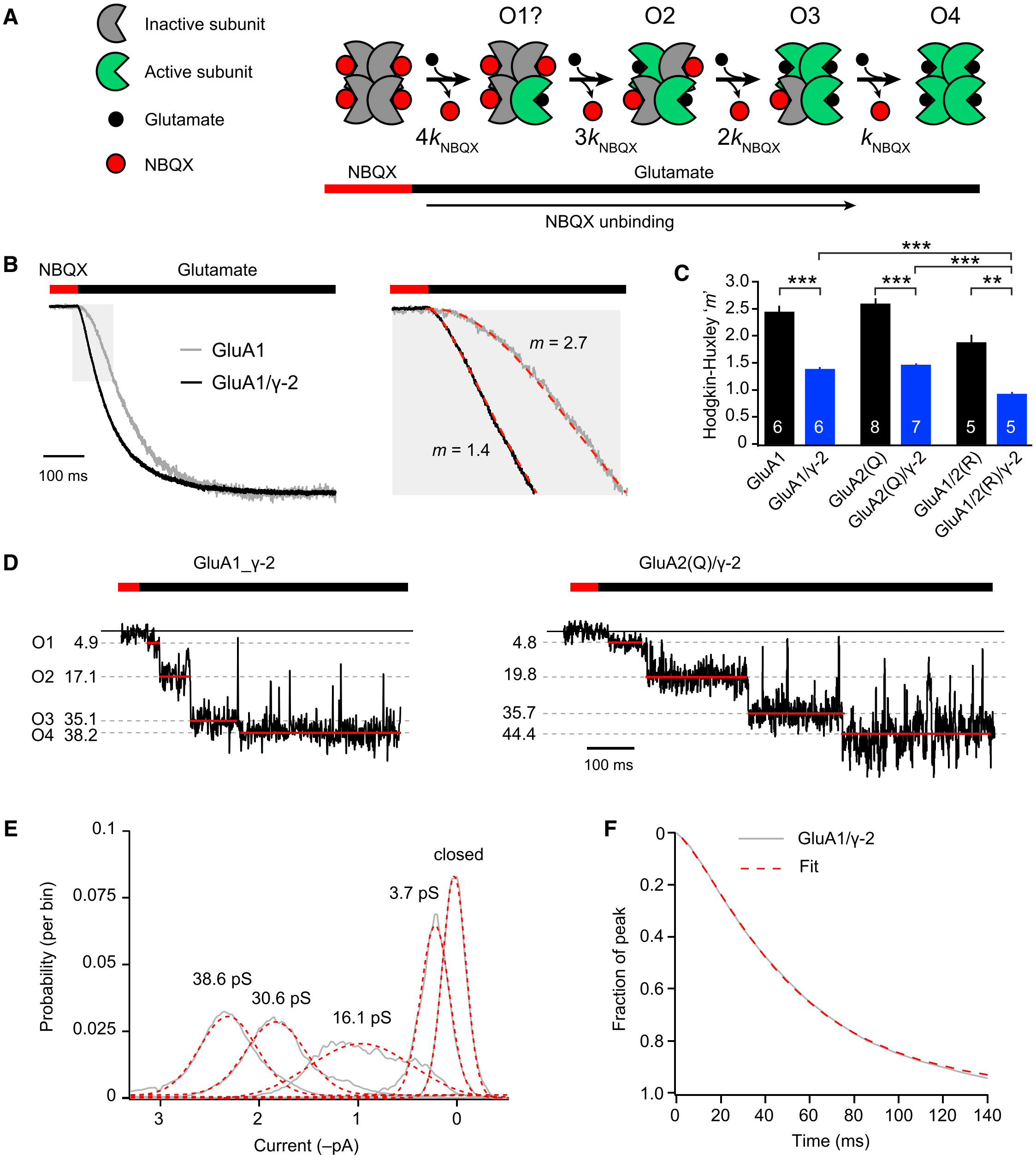

Figure 5. γ-2 Increases the Efficacy of Glutamate at Partially Liganded AMPARs

(A) Schematic of slow NBQX unbinding, which allows the time course of channel activation by glutamate to be observed.

(B) Normalized representative currents recorded following 50 μM NBQX/10 mM glutamate exchange (with 50 μM cyclothiazide). γ-2 speeds the current onset (enlargement of the highlighted section), reducing the Hodgkin-Huxley exponent (*m*) required to fit the records (dashed red lines).

(C) Pooled data from H-H fits. γ-2 accelerated the onset of NBQX/glutamate currents for each AMPAR subtype. Two-way ANOVA indicated significant main effects for AMPAR type ($F_{2,\,31}$ = 48.43, p < 0.0001) and for γ-2 ($F_{1,\,31}$ = 187.97, p < 0.0001) but no interaction between AMPAR type and TARP ($F_{2,\,31}$ = 0.41, p = 0.67). **p < 0.01, ***p < 0.001 (Welch t test). Symbols indicate mean and error bars indicate SEM.

(D) Representative single-channel NBQX/glutamate records displaying four discrete conductance levels. Measured conductance values are indicated.

(E) Normalized probability densities for closed, O1, O2, O3, and O4 conductance states, pooled from 560 identified conductances in 134 NBQX/glutamate sweeps. The dashed red lies are Gaussian fits.

(F) Normalized, pooled GluA1/γ-2 NBQX/glutamate current (n = 6). Data are fit (dashed red line) using the scheme shown in (A) (k_{NBQX}, 19 s^{-1}), with the mean estimates of the four conductance states (O1, 3.7 pS; O2, 16.1 pS; O3, 30.6 pS; O4, 38.6 pS).

See also Figure S2.

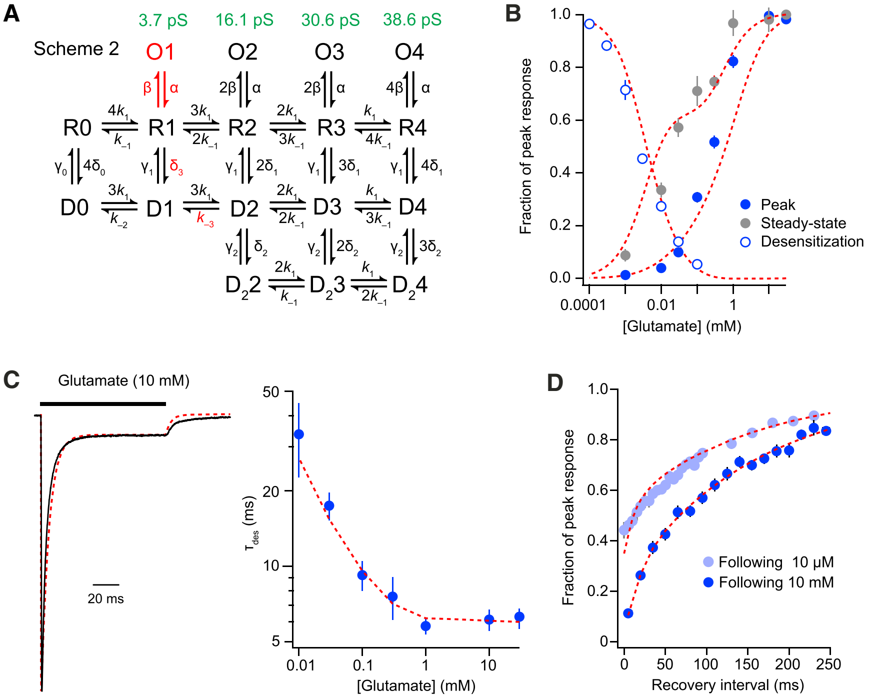

Figure 6. A Revised Kinetic Scheme Can Approximate Multiple Aspects of GluA1/γ-2 Behavior

(A) Scheme 2 is a modified form of scheme RH, with mechanistic changes highlighted (red) and assigned conductance levels (green).

(B–D) A single set of rates within scheme 2 can simultaneously approximate (dashed red lines) the three concentration response relationships (B), desensitization kinetics (C), and recovery kinetics (D) of GluA1/γ-2. Symbols indicate mean and error bars indicate SEM. The following rate constants were used: $k_1 = 1.3 \times 10^7\ M^{-1}s^{-1}$, $k_{-1} = 3{,}000\ s^{-1}$, $\alpha = 1{,}000\ s^{-1}$, $\beta = 6{,}000\ s^{-1}$, $\delta_1 = 1{,}200\ s^{-1}$, $\gamma_1 = 16\ s^{-1}$, $\delta_2 = 1{,}300\ s^{-1}$, $\gamma_2 = 3{,}900\ s^{-1}$, $\delta_3 = 250\ s^{-1}$, $\delta_0 = 0.48\ s^{-1}$, $\gamma_0 = 4.4\ s^{-1}$, $k_{-2} = 63\ s^{-1}$, and $k_{-3} = 630\ s^{-1}$. See also Figure S3.

make a greater contribution to currents from heteromeric GluA1/2(R) receptors than from homomeric GluA2(Q).

Kinetic Modeling of GluA1/γ-2

We next attempted to mimic our data by modifying the full kinetic scheme previously developed for GluA1 (scheme RH, Figure S3A; Robert and Howe, 2003). Using this scheme, and allowing the published rates to vary by ≤20%, we were able to replicate our GluA1 concentration-response curves for peak activation and desensitization as well as the observed kinetics of desensitization and recovery (Figures S3B–S3D).

To accommodate our GluA1/γ-2 data, we modified scheme RH. To reflect the TARP-dependent reduction in desensitization by low concentrations of glutamate, we decreased the rate of desensitization of mono-liganded TARPed receptors (R1 → D1). We also included an additional open state, O1, and assigned O1–O4 the measured subconductances from Figure 5. Finally, for improved estimation of our steady-state dose-response curves, we assigned doubly and triply liganded receptors equal opening rates. With these changes, our modified scheme (scheme 2, Figure 6A) was able to approximate all three concentration-response relationships, notably reducing the separation between desensitization sensitivity and channel activation, and replicating the shoulder of the steady-state data (Figure 6B). The same set of rate constants was also able to describe the kinetics of desensitization (Figure 6C) and recovery (Figure 6D) at multiple glutamate concentrations. Of note, modest changes to rate constants in our model were able to generate bell-shaped steady-state concentration-response curves (Morimoto-Tomita et al., 2009; Semenov et al., 2012; data not shown). Overall, scheme 2 is capable of accommodating and explaining key functional properties of GluA1/γ-2, including the biphasic concentration-response curves previously suggested to arise from the functional uncoupling of TARPs and AMPARs.

Modeling of Synaptic Currents

It seemed likely that the behavior we observed for GluA1/γ-2 at low glutamate concentrations would be expected to result in a decreased desensitization of synaptic receptors by prolonged low concentrations of transmitter of the sort that can occur during spillover (Carter and Regehr, 2000; DiGregorio et al., 2002; Nielsen et al., 2004) or delayed synaptic clearance (Trussell et al., 1993). Using either scheme RH (for GluA1) or scheme 2 (for GluA1/γ-2), we simulated brief receptor activations (two 1-ms pulses, 10 mM glutamate, 10-ms interval) with or without the continued presence of 1 μM glutamate. Scheme RH predicted that this concentration of background glutamate would inhibit GluA1-mediated charge transfer by 66%. By contrast, scheme 2 predicted only an 18% reduction in GluA1/γ-2-mediated charge transfer. These predictions were borne out by experiments in which 1 μM glutamate resulted in a 56% ± 3% inhibition of GluA1 (n = 4) but only 14% ± 5% inhibition of GluA1/γ-2 (n = 5, p = 0.00039) (Figures 7A–7C).

Having demonstrated that γ-2 reduces the inhibitory effects of background glutamate on GluA1 charge transfer and that scheme 2 is capable of replicating these properties, we next used scheme RH and scheme 2 to model GluA1 and GluA1/γ-2 responses to glutamate spillover waveforms. To this end, as an exemplar, we used a previously developed diffusion model of the cerebellar mossy fiber to granule cell (MF-GC) synapse (Nielsen et al., 2004), allowing us to model AMPAR activation by local vesicular release or spillover from distant release sites (DiGregorio et al., 2002). We first modeled the influence of γ-2 on responses to single-vesicle fusion events (Figure 7D). As expected, the peak channel conductance following local release was increased by γ-2, and the total charge transfer (measured over 3 ms) was ~5-fold larger than that modeled with GluA1 alone. However, when we simulated the response to vesicle fusion at the nearest neighboring synapse, charge transfer was

OPEN
ACCESS
CellPress

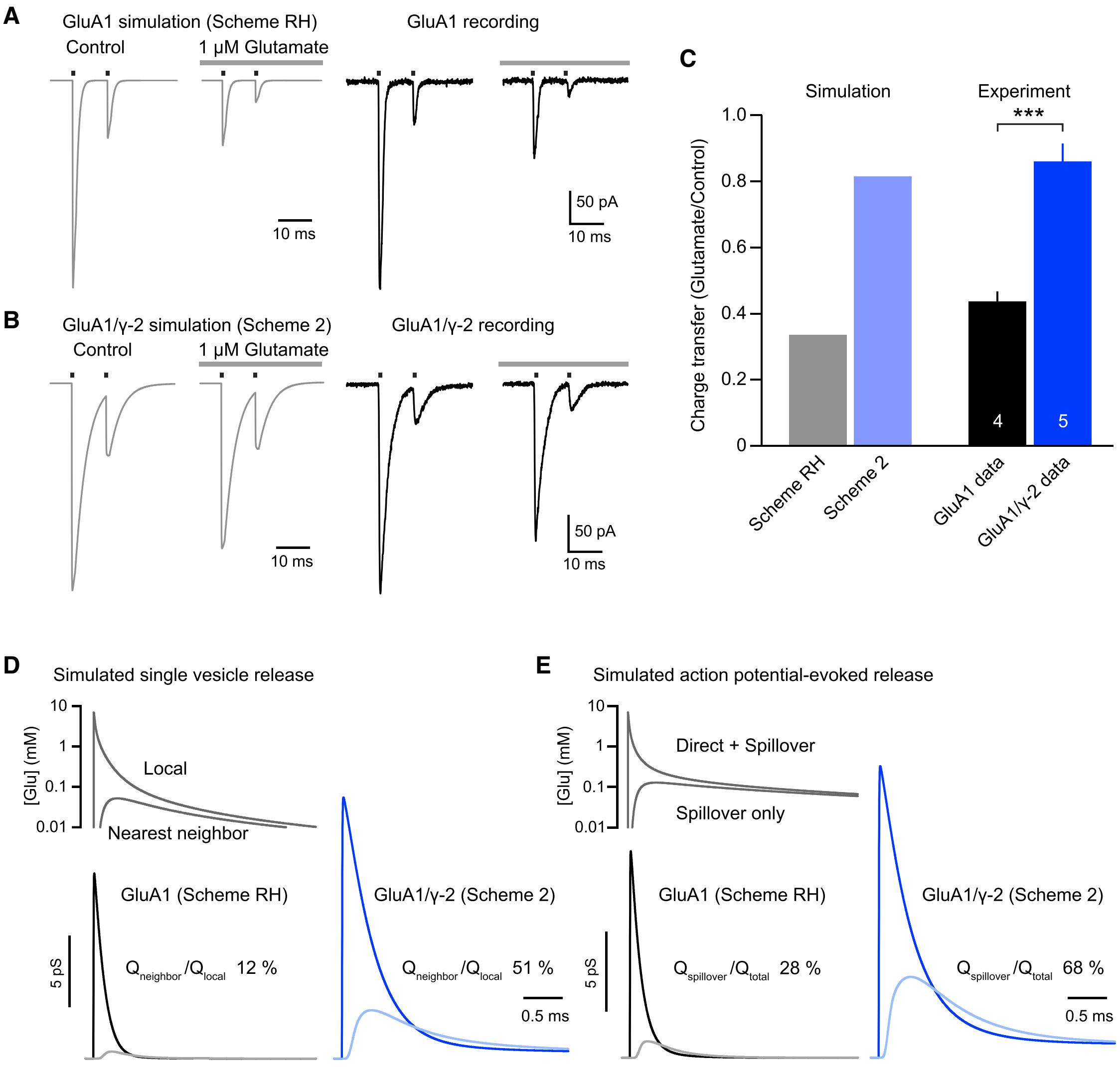

Figure 7. Co-expression of γ-2 Reduces Inhibition of GluA1-Mediated Charge Transfer by Background Glutamate and Is Predicted to Enhance Charge Transfer by Spillover

(A) Simulated responses of GluA1 (scheme RH, left) and recorded GluA1 currents (right) in response to two 1-ms pulses of 10 mM glutamate (100 Hz), with and without background application of 1 μM glutamate. Charge transfer was measured as the area under the curve.

(B) As for (A) but for GluA1/γ-2, simulated using scheme 2, and for GluA1/γ-2 currents.

(C) Bar chart showing that simulations and data are in agreement, with ~4-fold less glutamate-mediated reduction in charge transfer for γ-2-containing receptors than for GluA1 alone. Bars indicate mean and error bars indicate SEM.

(D and E) Responses of GluA1 (scheme RH) and GluA1/γ-2 (scheme 2) to simulated synaptic glutamate waveforms (Nielsen et al., 2004). The concentration profiles used represent (D) fusion of a single vesicle either locally or at the nearest-neighbor active zone or (E) action potential-evoked release causing prolonged spillover, either with or without local release. Spillover or nearest-neighbor charge as a percentage of direct or local charge is indicated. ***$p > 0.001$ (Welch t test). See also Figure S4.

increased >20-fold by γ-2. Indeed, for GluA1/γ-2, the total predicted charge transfer from nearest-neighbor release was over half of that elicited by local release.

We next simulated GluA1 and GluA1/γ-2 responses to evoked release. The MF-GC synaptic model consists of a 7 × 7 array of synaptic connections, with the central synapse being monitored, and, following an action potential, the probability of vesicular release at any one site is 0.46 (Nielsen et al., 2004). We simulated two glutamate waveforms: with the presence of local release (direct + spillover) and in the absence of local release (spillover only). Again, γ-2 greatly influenced the predicted response, increasing total charge transfer by >5-fold for direct activation but by 12-fold for spillover only. In this case, GluA1/γ-2 spillover-mediated charge transfer was predicted to reach nearly 70% of that resulting from direct activation. For both local and evoked release, increased charge transfer in the presence of γ-2 is to be expected, given the known action of TARPs on AMPAR conductance and deactivation. The dual effects of

TARPs on the desensitization and conductance of singly occupied AMPARs make only a limited contribution to the increase in charge following direct release (<10%), but they contribute ~40% of charge transfer for evoked spillover currents and ~65% of charge transfer from nearest-neighbor single-vesicle release (Figure S4). Accordingly, our findings suggest that the behavior of synaptic receptors during glutamate spillover will be profoundly influenced by the presence of TARPs.

DISCUSSION

One of the canonical properties of TARPs is their ability to reduce AMPAR desensitization (Priel et al., 2005; Tomita et al., 2005; Turetsky et al., 2005). Surprisingly, despite the importance of desensitization during spillover and delayed clearance of transmitter following an excitatory postsynaptic current (EPSC) (DiGregorio et al., 2007; Trussell et al., 1993), the influence of TARPs on AMPAR desensitization at low concentrations of glutamate has not been described previously. By determining the glutamate concentration dependence of TARP action, we have revealed three fundamental features of AMPAR behavior. First, we find that γ-2 induces opposite shifts in glutamate potency for AMPAR desensitization (~5-fold increase in IC_{50}) and activation (~4-fold reduction in EC_{50}). Second, for γ-2-containing receptors, we find a marked concentration dependence of entry into, but not recovery from, desensitization, revealing a TARP-induced increase in the steady-state efficacy of low concentrations of glutamate. Third, we find that γ-2 also enhances the efficacy of glutamate by promoting the opening of singly occupied receptors. Together, our data indicate that γ-2 shifts the balance of GluA1 gating at low agonist concentrations from desensitization to activation. Our experiments and simulations suggest that this altered AMPAR gating, rather than a functional uncoupling of TARPs, is likely to account for the phenomenon of autoinactivation. Moreover, we propose that this TARP-dependent behavior at low glutamate concentrations will greatly enhance the response of AMPARs during transmitter spillover.

Using the kinetic scheme of Robert and Howe (2003), we were able to replicate our concentration response and kinetic data by introducing an open state for the singly occupied receptor, slowing its rate of entry into the desensitized state (R1 → D1), and incorporating the occupancy-dependent conductance values from our single-channel patches. However, this scheme does not generate "superactivation," a slow "run-up" of AMPAR/TARP currents on a timescale of ~1 s (Carbone and Plested, 2016). As with autoinactivation, superactivation has been proposed to result from a state dependence of the functional interaction between AMPAR and TARP. This is potentially reflected in single-channel records as a high open probability "mode" (Zhang et al., 2014) that contributes to "steady-state" currents seen during trains of glutamate application (Devi et al., 2016). We did not observe superactivation in our recordings, perhaps because the phenomenon appears to be less pronounced for γ-2 than for other TARPs (Kato et al., 2010) and is most evident when AMPARs are saturated with TARPs (Carbone and Plested, 2016).

Our model accommodates all of our experimental data and suggests an alternative explanation for the phenomenon of autoinactivation that does not require functional uncoupling of AMPAR and TARP. Of note, markedly biphasic steady-state concentration-response curves are characteristic of kainate receptors (KARs), fellow members of the ionotropic glutamate receptor (iGluR) superfamily, and occur independent of auxiliary subunits. For heteromeric KARs containing low-affinity (GluK1–3) and high-affinity (GluK4–5) subunits, only the high-affinity subunit of each LBD dimer is occupied at low glutamate concentrations, and this is sufficient to cause channel openings of maximal conductance (Mott et al., 2010; Smith and Howe, 2000) but not receptor desensitization (Fisher and Mott, 2011; Mott et al., 2010). Only at high glutamate concentrations, when the low-affinity subunit is also occupied, can desensitization be triggered. Although the full gating and minimal desensitization at low occupancy are more pronounced for KARs, we propose that TARPed AMPARs behave in a comparable manner.

Using three different approaches we found no evidence for AMPAR/TARP uncoupling. However, we cannot exclude that desensitized AMPARs (for example, those in state D4) are functionally or physically uncoupled from TARPs but then rapidly re-associate (in state R4). Nevertheless, biochemical evidence supporting the concept of functional uncoupling—AMPAR agonist-triggered reduction in AMPAR and TARP co-immunoprecipitation (Morimoto-Tomita et al., 2009; Tomita et al., 2004)—was not observed in other studies (Nakagawa et al., 2005; Semenov et al., 2012). Further, although three different AMPAR_TARP tandem constructs were originally shown not to autoinactivate (Morimoto-Tomita et al., 2009), a subsequent study observed autoinactivation of the GluA4_γ-2 tandem, suggesting that this phenomenon can occur in the absence of physical dissociation (Semenov et al., 2012).

Recent cryoelectron microscopy (cryo-EM) structures of GluA2/γ-2 reveal extensive intra-membrane contacts between AMPAR and TARP (Twomey et al., 2016; Zhao et al., 2016), and these appear to be important both for AMPAR/TARP assembly and function (Ben-Yaacov et al., 2017). Although the cryo-EM structures are of channels in their closed states, the conformation of AMPAR transmembrane regions are predicted to be similar following desensitization (Dong and Zhou, 2011; Dürr et al., 2014; Meyerson et al., 2014; Sobolevsky et al., 2009). Therefore, one might expect that, in the desensitized state, TARPs maintain a close association with the AMPAR. By contrast, the AMPAR extracellular domains undergo large rotational rearrangements following desensitization (Dürr et al., 2014; Herguedas et al., 2016; Meyerson et al., 2014), which would be expected to break the charge-mediated interactions between the AMPAR LBD and TARP Ex1 (Dawe et al., 2016; Twomey et al., 2016; Zhao et al., 2016) in at least one subunit. Even so, although alteration of the charges in the LBD has been shown to greatly diminish TARP-induced slowing of deactivation and desensitization, other TARP-associated effects persisted (increased kainate efficacy and decreased block by intracellular spermine) (Dawe et al., 2016), suggesting that, even when LBD/Ex1 interaction is eliminated, TARPs and AMPARs remain functionally coupled.

Our results suggest that the dual effects of TARPs on glutamate efficacy will have the greatest effect on native receptors during prolonged exposure to low concentrations of glutamate, as occurs, for example, during transmitter spillover (Carter and

Regehr, 2000; DiGregorio et al., 2002, 2007; Nielsen et al., 2004), delayed synaptic clearance (Kinney et al., 1997; Otis et al., 1996; Trussell et al., 1993; Zampini et al., 2016), or volume transmission (Szapiro and Barbour, 2007). Specifically, TARP-associated AMPARs will be able to pass appreciable current when exposed to low-micromolar glutamate and will remain responsive to high concentrations of glutamate resulting from vesicular release. Our synaptic simulations demonstrate that γ-2 imparts a marked resistance to desensitization by glutamate spillover and allows significant postsynaptic responses even in the absence of local release. Generalizing this action of γ-2 to the GluA2/4 heteromers present in cerebellar granule cells would account for the large steady-state currents generated by synaptic AMPARs in these cells (DiGregorio et al., 2007). Activation of AMPARs via glutamate spillover accounts for the majority of the charge injected into granule cells during high-frequency mossy fiber stimulation (Saviane and Silver, 2006) and underlies the primary excitatory drive of granule cells during locomotion (Powell et al., 2015). Thus, resistance to desensitization of TARP-associated AMPARs appears to be key for synaptic signaling in the input layer of the cerebellum and is likely important at other sites where glutamate spillover occurs.

EXPERIMENTAL PROCEDURES

Heterologous Expression

HEK293 cells were transfected with recombinant AMPAR subunits and TARPs (plus EGFP). AMPAR subunit cDNAs (rat) were “flip” splice variants, and the GluA2 forms were additionally arginine/glycine (R/G)-edited. The GluA1_γ-2 tandem consisted of full-length GluA1 and a nine-amino acid linker (GGGG GEFAT) before the start codon of full-length γ-2. For further details, see the Supplemental Experimental Procedures.

Rapid Agonist Application to Excised Patches

Voltage-clamp recordings were made from outside-out patches. Rapid agonist application was achieved by switching between continuously flowing solutions using piezoelectric translation of an application tool made from either theta glass or custom triple-barreled glass, as described in the Supplemental Experimental Procedures.

Data Analysis and Kinetic Modeling

Records were analyzed using Igor Pro 6.35 (Wavemetrics) with Neuromatic 2.8 (http://www.neuromatic.thinkrandom.com). Kinetic simulations were performed in Scilab 5.5.0. (Scilab Enterprises; http://www.scilab.org). For further details, see the Supplemental Experimental Procedures.

Data Presentation and Statistical Analysis

Summary data are presented in the text as mean ± SEM (from *n* patches). Comparisons involving two datasets only were performed using a two-sided Welch two-sample t test that did not assume equal variance (normality was not tested statistically but gauged from quantile-quantile [Q-Q] plots and/or density histograms). Analyses involving data from three or more groups were performed using one- or two-way ANOVA (Welch heteroscedastic *F* test), followed by pairwise comparisons using two-sided Welch two-sample t tests with Bonferroni correction where appropriate. Differences were considered significant at $p < 0.05$. Exact *p* values are presented to two significant figures, except when $p < 0.0001$. Differences were considered significant at $p < 0.05$. Statistical tests were performed using R (3.3.2, the R Foundation for Statistical Computing; http://www.r-project.org/) and R Studio (1.0.143, RStudio). No statistical test was used to predetermine sample sizes; these were based on standards of the field. No randomization was used.

SUPPLEMENTAL INFORMATION

Supplemental Information includes Supplemental Experimental Procedures and four figures and can be found with this article online at http://dx.doi.org/10.1016/j.celrep.2017.07.014.

AUTHOR CONTRIBUTIONS

I.D.C., D.M.M., M.F., and S.G.C.C. designed the experiments. I.D.C. and D.M.M. performed the experiments. I.D.C., D.M.M., and M.F. analyzed the data. I.D.C., D.M.M., V.J., M.F., and S.G.C.C. interpreted the results. I.D.C. and M.F. prepared the figures and wrote the manuscript with input from all authors.

ACKNOWLEDGMENTS

This work was supported by the MRC (MR/J002976/1 to S.G.C.C. and M.F. and MR/J012998/1 to M.F. and S.G.C.C.), the Wellcome Trust (086185/Z/08/Z to S.G.C.C. and M.F.), and the NIH (K99NS094761 to D.M.M. and R01 GM113212 and 1R35GM122528 to V.J.). We thank Jason Rothman for assistance with diffusion models and Angus Silver for comments on the manuscript.

Received: March 23, 2017
Revised: June 12, 2017
Accepted: July 9, 2017
Published: August 1, 2017

REFERENCES

Armstrong, N., and Gouaux, E. (2000). Mechanisms for activation and antagonism of an AMPA-sensitive glutamate receptor: crystal structures of the GluR2 ligand binding core. Neuron *28*, 165–181.

Armstrong, N., Jasti, J., Beich-Frandsen, M., and Gouaux, E. (2006). Measurement of conformational changes accompanying desensitization in an ionotropic glutamate receptor. Cell *127*, 85–97.

Barbour, B., and Häusser, M. (1997). Intersynaptic diffusion of neurotransmitter. Trends Neurosci. *20*, 377–384.

Ben-Yaacov, A., Gillor, M., Haham, T., Parsai, A., Qneibi, M., and Stern-Bach, Y. (2017). Molecular mechanism of AMPA receptor modulation by TARP/stargazin. Neuron *93*, 1126–1137.e4.

Boulter, J., Hollmann, M., O’Shea-Greenfield, A., Hartley, M., Deneris, E., Maron, C., and Heinemann, S. (1990). Molecular cloning and functional expression of glutamate receptor subunit genes. Science *249*, 1033–1037.

Budisantoso, T., Harada, H., Kamasawa, N., Fukazawa, Y., Shigemoto, R., and Matsui, K. (2013). Evaluation of glutamate concentration transient in the synaptic cleft of the rat calyx of Held. J. Physiol. *591*, 219–239.

Cais, O., Herguedas, B., Krol, K., Cull-Candy, S.G., Farrant, M., and Greger, I.H. (2014). Mapping the interaction sites between AMPA receptors and TARPs reveals a role for the receptor N-terminal domain in channel gating. Cell Rep. *9*, 728–740.

Carbone, A.L., and Plested, A.J. (2016). Superactivation of AMPA receptors by auxiliary proteins. Nat. Commun. *7*, 10178.

Carter, A.G., and Regehr, W.G. (2000). Prolonged synaptic currents and glutamate spillover at the parallel fiber to stellate cell synapse. J. Neurosci. *20*, 4423–4434.

Cho, C.H., St-Gelais, F., Zhang, W., Tomita, S., and Howe, J.R. (2007). Two families of TARP isoforms that have distinct effects on the kinetic properties of AMPA receptors and synaptic currents. Neuron *55*, 890–904.

Clements, J.D., Lester, R.A., Tong, G., Jahr, C.E., and Westbrook, G.L. (1992). The time course of glutamate in the synaptic cleft. Science *258*, 1498–1501.

Clements, J.D., Feltz, A., Sahara, Y., and Westbrook, G.L. (1998). Activation kinetics of AMPA receptor channels reveal the number of functional agonist binding sites. J. Neurosci. *18*, 119–127.

Constals, A., Penn, A.C., Compans, B., Toulmé, E., Phillipat, A., Marais, S., Retailleau, N., Hafner, A.S., Coussen, F., Hosy, E., and Choquet, D. (2015). Glutamate-induced AMPA receptor desensitization increases their mobility and modulates short-term plasticity through unbinding from Stargazin. Neuron *85*, 787–803.

Dawe, G.B., Musgaard, M., Aurousseau, M.R., Nayeem, N., Green, T., Biggin, P.C., and Bowie, D. (2016). Distinct structural pathways coordinate the activation of AMPA receptor-auxiliary subunit complexes. Neuron *89*, 1264–1276.

Devi, S.P., Howe, J.R., and Auger, C. (2016). Train stimulation of parallel fibre to Purkinje cell inputs reveals two populations of synaptic responses with different receptor signatures. J. Physiol. *594*, 3705–3727.

DiGregorio, D.A., Nusser, Z., and Silver, R.A. (2002). Spillover of glutamate onto synaptic AMPA receptors enhances fast transmission at a cerebellar synapse. Neuron *35*, 521–533.

DiGregorio, D.A., Rothman, J.S., Nielsen, T.A., and Silver, R.A. (2007). Desensitization properties of AMPA receptors at the cerebellar mossy fiber granule cell synapse. J. Neurosci. *27*, 8344–8357.

Dong, H., and Zhou, H.X. (2011). Atomistic mechanism for the activation and desensitization of an AMPA-subtype glutamate receptor. Nat. Commun. *2*, 354.

Dürr, K.L., Chen, L., Stein, R.A., De Zorzi, R., Folea, I.M., Walz, T., Mchaourab, H.S., and Gouaux, E. (2014). Structure and dynamics of AMPA receptor GluA2 in resting, pre-open, and desensitized states. Cell *158*, 778–792.

Fisher, J.L., and Mott, D.D. (2011). Distinct functional roles of subunits within the heteromeric kainate receptor. J. Neurosci. *31*, 17113–17122.

Gebhardt, C., and Cull-Candy, S.G. (2006). Influence of agonist concentration on AMPA and kainate channels in CA1 pyramidal cells in rat hippocampal slices. J. Physiol. *573*, 371–394.

Geiger, J.R., Melcher, T., Koh, D.S., Sakmann, B., Seeburg, P.H., Jonas, P., and Monyer, H. (1995). Relative abundance of subunit mRNAs determines gating and Ca2+ permeability of AMPA receptors in principal neurons and interneurons in rat CNS. Neuron *15*, 193–204.

Hastie, P., Ulbrich, M.H., Wang, H.L., Arant, R.J., Lau, A.G., Zhang, Z., Isacoff, E.Y., and Chen, L. (2013). AMPA receptor/TARP stoichiometry visualized by single-molecule subunit counting. Proc. Natl. Acad. Sci. USA *110*, 5163–5168.

Heine, M., Groc, L., Frischknecht, R., Béïque, J.C., Lounis, B., Rumbaugh, G., Huganir, R.L., Cognet, L., and Choquet, D. (2008). Surface mobility of postsynaptic AMPARs tunes synaptic transmission. Science *320*, 201–205.

Henley, J.M., and Wilkinson, K.A. (2016). Synaptic AMPA receptor composition in development, plasticity and disease. Nat. Rev. Neurosci. *17*, 337–350.

Herguedas, B., García-Nafría, J., Cais, O., Fernández-Leiro, R., Krieger, J., Ho, H., and Greger, I.H. (2016). Structure and organization of heteromeric AMPA-type glutamate receptors. Science *352*, aad3873.

Jonas, P. (2000). The time course of signaling at central glutamatergic synapses. News Physiol. Sci. *15*, 83–89.

Kato, A.S., Gill, M.B., Ho, M.T., Yu, H., Tu, Y., Siuda, E.R., Wang, H., Qian, Y.W., Nisenbaum, E.S., Tomita, S., and Bredt, D.S. (2010). Hippocampal AMPA receptor gating controlled by both TARP and cornichon proteins. Neuron *68*, 1082–1096.

Kazi, R., Dai, J., Sweeney, C., Zhou, H.X., and Wollmuth, L.P. (2014). Mechanical coupling maintains the fidelity of NMDA receptor-mediated currents. Nat. Neurosci. *17*, 914–922.

Keinänen, K., Wisden, W., Sommer, B., Werner, P., Herb, A., Verdoorn, T.A., Sakmann, B., and Seeburg, P.H. (1990). A family of AMPA-selective glutamate receptors. Science *249*, 556–560.

Kim, K.S., Yan, D., and Tomita, S. (2010). Assembly and stoichiometry of the AMPA receptor and transmembrane AMPA receptor regulatory protein complex. J. Neurosci. *30*, 1064–1072.

Kinney, G.A., Overstreet, L.S., and Slater, N.T. (1997). Prolonged physiological entrapment of glutamate in the synaptic cleft of cerebellar unipolar brush cells. J. Neurophysiol. *78*, 1320–1333.

Landes, C.F., Rambhadran, A., Taylor, J.N., Salatan, F., and Jayaraman, V. (2011). Structural landscape of isolated agonist-binding domains from single AMPA receptors. Nat. Chem. Biol. *7*, 168–173.

Lomeli, H., Mosbacher, J., Melcher, T., Höger, T., Geiger, J.R., Kuner, T., Monyer, H., Higuchi, M., Bach, A., and Seeburg, P.H. (1994). Control of kinetic properties of AMPA receptor channels by nuclear RNA editing. Science *266*, 1709–1713.

MacLean, D.M. (2013). CrossTalk opposing view: TARPs modulate AMPA receptor conformations before the gating transitions. J. Physiol. *591*, 1585–1586, discussion 1587.

MacLean, D.M., Ramaswamy, S.S., Du, M., Howe, J.R., and Jayaraman, V. (2014). Stargazin promotes closure of the AMPA receptor ligand-binding domain. J. Gen. Physiol. *144*, 503–512.

Mayer, M.L. (2016). Structural biology of glutamate receptor ion channel complexes. Curr. Opin. Struct. Biol. *41*, 119–127.

Meyerson, J.R., Kumar, J., Chittori, S., Rao, P., Pierson, J., Bartesaghi, A., Mayer, M.L., and Subramaniam, S. (2014). Structural mechanism of glutamate receptor activation and desensitization. Nature *514*, 328–334.

Milstein, A.D., Zhou, W., Karimzadegan, S., Bredt, D.S., and Nicoll, R.A. (2007). TARP subtypes differentially and dose-dependently control synaptic AMPA receptor gating. Neuron *55*, 905–918.

Morimoto-Tomita, M., Zhang, W., Straub, C., Cho, C.H., Kim, K.S., Howe, J.R., and Tomita, S. (2009). Autoinactivation of neuronal AMPA receptors via glutamate-regulated TARP interaction. Neuron *61*, 101–112.

Mott, D.D., Rojas, A., Fisher, J.L., Dingledine, R.J., and Benveniste, M. (2010). Subunit-specific desensitization of heteromeric kainate receptors. J. Physiol. *588*, 683–700.

Nakagawa, T., Cheng, Y., Ramm, E., Sheng, M., and Walz, T. (2005). Structure and different conformational states of native AMPA receptor complexes. Nature *433*, 545–549.

Nielsen, T.A., DiGregorio, D.A., and Silver, R.A. (2004). Modulation of glutamate mobility reveals the mechanism underlying slow-rising AMPAR EPSCs and the diffusion coefficient in the synaptic cleft. Neuron *42*, 757–771.

Otis, T., Zhang, S., and Trussell, L.O. (1996). Direct measurement of AMPA receptor desensitization induced by glutamatergic synaptic transmission. J. Neurosci. *16*, 7496–7504.

Partin, K.M., Fleck, M.W., and Mayer, M.L. (1996). AMPA receptor flip/flop mutants affecting deactivation, desensitization, and modulation by cyclothiazide, aniracetam, and thiocyanate. J. Neurosci. *16*, 6634–6647.

Powell, K., Mathy, A., Duguid, I., and Häusser, M. (2015). Synaptic representation of locomotion in single cerebellar granule cells. eLife *4*, e07290.

Priel, A., Kolleker, A., Ayalon, G., Gillor, M., Osten, P., and Stern-Bach, Y. (2005). Stargazin reduces desensitization and slows deactivation of the AMPA-type glutamate receptors. J. Neurosci. *25*, 2682–2686.

Raman, I.M., and Trussell, L.O. (1992). The kinetics of the response to glutamate and kainate in neurons of the avian cochlear nucleus. Neuron *9*, 173–186.

Ramaswamy, S., Cooper, D., Poddar, N., MacLean, D.M., Rambhadran, A., Taylor, J.N., Uhm, H., Landes, C.F., and Jayaraman, V. (2012). Role of conformational dynamics in α-amino-3-hydroxy-5-methylisoxazole-4-propionic acid (AMPA) receptor partial agonism. J. Biol. Chem. *287*, 43557–43564.

Robert, A., and Howe, J.R. (2003). How AMPA receptor desensitization depends on receptor occupancy. J. Neurosci. *23*, 847–858.

Rosenmund, C., Stern-Bach, Y., and Stevens, C.F. (1998). The tetrameric structure of a glutamate receptor channel. Science *280*, 1596–1599.

Saviane, C., and Silver, R.A. (2006). Fast vesicle reloading and a large pool sustain high bandwidth transmission at a central synapse. Nature *439*, 983–987.

Schwenk, J., Harmel, N., Zolles, G., Bildl, W., Kulik, A., Heimrich, B., Chisaka, O., Jonas, P., Schulte, U., Fakler, B., and Klöcker, N. (2009). Functional proteomics identify cornichon proteins as auxiliary subunits of AMPA receptors. Science *323*, 1313–1319.

Schwenk, J., Harmel, N., Brechet, A., Zolles, G., Berkefeld, H., Müller, C.S., Bildl, W., Baehrens, D., Hüber, B., Kulik, A., et al. (2012). High-resolution proteomics unravel architecture and molecular diversity of native AMPA receptor complexes. Neuron *74*, 621–633.

Semenov, A., Möykkynen, T., Coleman, S.K., Korpi, E.R., and Keinänen, K. (2012). Autoinactivation of the stargazin-AMPA receptor complex: subunit-dependency and independence from physical dissociation. PLoS ONE *7*, e49282.

Shaikh, S.A., Dolino, D.M., Lee, G., Chatterjee, S., MacLean, D.M., Flatebo, C., Landes, C.F., and Jayaraman, V. (2016). Stargazin modulation of AMPA receptors. Cell Rep. *17*, 328–335.

Shanks, N.F., Savas, J.N., Maruo, T., Cais, O., Hirao, A., Oe, S., Ghosh, A., Noda, Y., Greger, I.H., Yates, J.R., 3rd, and Nakagawa, T. (2012). Differences in AMPA and kainate receptor interactomes facilitate identification of AMPA receptor auxiliary subunit GSG1L. Cell Rep. *1*, 590–598.

Shi, Y., Lu, W., Milstein, A.D., and Nicoll, R.A. (2009). The stoichiometry of AMPA receptors and TARPs varies by neuronal cell type. Neuron *62*, 633–640.

Smith, T.C., and Howe, J.R. (2000). Concentration-dependent substate behavior of native AMPA receptors. Nat. Neurosci. *3*, 992–997.

Sobolevsky, A.I., Rosconi, M.P., and Gouaux, E. (2009). X-ray structure, symmetry and mechanism of an AMPA-subtype glutamate receptor. Nature *462*, 745–756.

Soto, D., Coombs, I.D., Kelly, L., Farrant, M., and Cull-Candy, S.G. (2007). Stargazin attenuates intracellular polyamine block of calcium-permeable AMPA receptors. Nat. Neurosci. *10*, 1260–1267.

Soto, D., Coombs, I.D., Renzi, M., Zonouzi, M., Farrant, M., and Cull-Candy, S.G. (2009). Selective regulation of long-form calcium-permeable AMPA receptors by an atypical TARP, gamma-5. Nat. Neurosci. *12*, 277–285.

Soto, D., Coombs, I.D., Gratacòs-Batlle, E., Farrant, M., and Cull-Candy, S.G (2014). Molecular mechanisms contributing to TARP regulation of channel conductance and polyamine block of calcium-permeable AMPA receptors. J. Neurosci. *34*, 11673–11683.

Sun, Y., Olson, R., Horning, M., Armstrong, N., Mayer, M., and Gouaux, E. (2002). Mechanism of glutamate receptor desensitization. Nature *417*, 245–253.

Suzuki, E., Kessler, M., and Arai, A.C. (2008). The fast kinetics of AMPA GluR3 receptors is selectively modulated by the TARPs gamma 4 and gamma 8. Mol. Cell. Neurosci. *38*, 117–123.

Szapiro, G., and Barbour, B. (2007). Multiple climbing fibers signal to molecular layer interneurons exclusively via glutamate spillover. Nat. Neurosci. *10*, 735–742.

Tomita, S. (2010). Regulation of ionotropic glutamate receptors by their auxiliary subunits. Physiology (Bethesda) *25*, 41–49.

Tomita, S., Fukata, M., Nicoll, R.A., and Bredt, D.S. (2004). Dynamic interaction of stargazin-like TARPs with cycling AMPA receptors at synapses. Science *303*, 1508–1511.

Tomita, S., Adesnik, H., Sekiguchi, M., Zhang, W., Wada, K., Howe, J.R., Nicoll, R.A., and Bredt, D.S. (2005). Stargazin modulates AMPA receptor gating and trafficking by distinct domains. Nature *435*, 1052–1058.

Traynelis, S.F., Wollmuth, L.P., McBain, C.J., Menniti, F.S., Vance, K.M., Ogden, K.K., Hansen, K.B., Yuan, H., Myers, S.J., and Dingledine, R. (2010). Glutamate receptor ion channels: structure, regulation, and function. Pharmacol. Rev. *62*, 405–496.

Trussell, L.O., Zhang, S., and Raman, I.M. (1993). Desensitization of AMPA receptors upon multiquantal neurotransmitter release. Neuron *10*, 1185–1196.

Turetsky, D., Garringer, E., and Patneau, D.K. (2005). Stargazin modulates native AMPA receptor functional properties by two distinct mechanisms. J. Neurosci. *25*, 7438–7448.

Twomey, E.C., Yelshanskaya, M.V., Grassucci, R.A., Frank, J., and Sobolevsky, A.I. (2016). Elucidation of AMPA receptor-stargazin complexes by cryo-electron microscopy. Science *353*, 83–86.

von Engelhardt, J., Mack, V., Sprengel, R., Kavenstock, N., Li, K.W., Stern-Bach, Y., Smit, A.B., Seeburg, P.H., and Monyer, H. (2010). CKAMP44: a brain-specific protein attenuating short-term synaptic plasticity in the dentate gyrus. Science *327*, 1518–1522.

Zampini, V., Liu, J.K., Diana, M.A., Maldonado, P.P., Brunel, N., and Dieudonné, S. (2016). Mechanisms and functional roles of glutamatergic synapse diversity in a cerebellar circuit. eLife *5*, e15872.

Zhang, W., Cho, Y., Lolis, E., and Howe, J.R. (2008). Structural and single-channel results indicate that the rates of ligand binding domain closing and opening directly impact AMPA receptor gating. J. Neurosci. *28*, 932–943.

Zhang, W., Devi, S.P., Tomita, S., and Howe, J.R. (2014). Auxiliary proteins promote modal gating of AMPA- and kainate-type glutamate receptors. Eur. J. Neurosci. *39*, 1138–1147.

Zhao, Y., Chen, S., Yoshioka, C., Baconguis, I., and Gouaux, E. (2016). Architecture of fully occupied GluA2 AMPA receptor-TARP complex elucidated by cryo-EM. Nature *536*, 108–111.

Cell Reports, Volume *20*

Supplemental Information

Dual Effects of TARP γ-2 on Glutamate Efficacy

Can Account for AMPA Receptor Autoinactivation

Ian D. Coombs, David M. MacLean, Vasanthi Jayaraman, Mark Farrant, and Stuart G. Cull-Candy

FIGURE S1

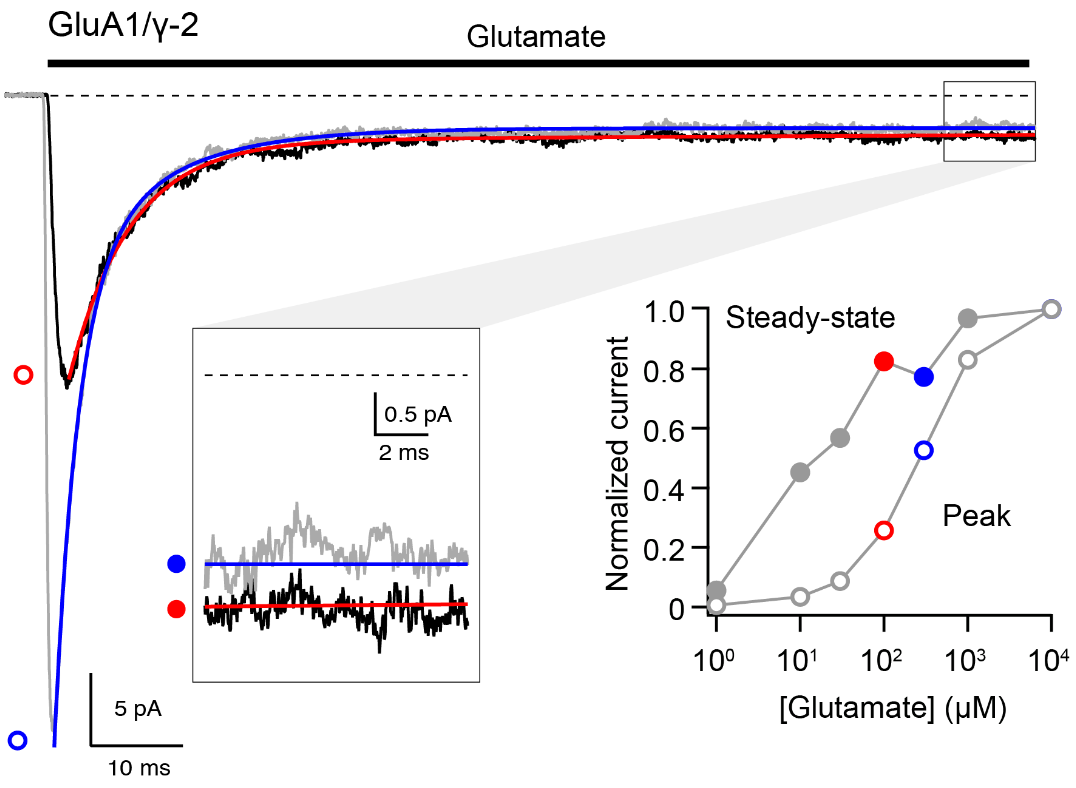

Figure S1 (related to Figure 1). Enhanced steady-state GluA1/γ-2 current at 100 versus 300 μM glutamate underlies the autoinactivation of the steady-state concentration response curve.
GluA1/γ-2 currents elicited by fast application of 100 μM glutamate (black record, red biexponential fit) and 300 μM glutamate (grey record, blue fit). Left-hand inset highlights the inverted concentration dependence of the steady-state current. Right-hand inset shows the contribution of these two records to both concentration-response relationships.

FIGURE S2

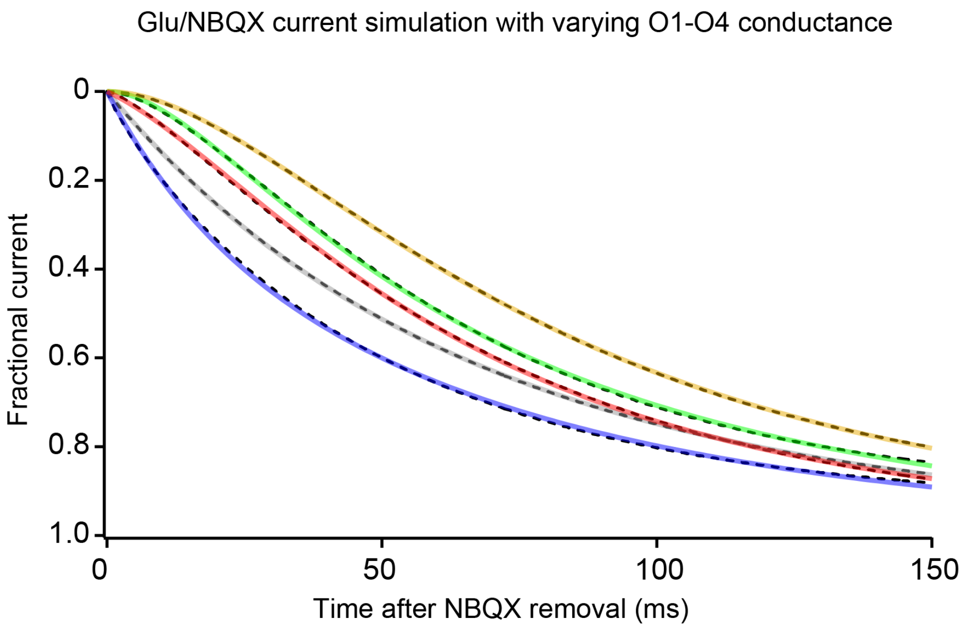

	Conductance (pS) O1	O2	O3	O4	'm'	
yellow	0	5	15	23	1.97	(Rosenmund et al.)
green	0	9	15	21	1.77	(Robert and Howe)
red	3.7	16.1	30.6	38.6	1.38	(Figure 5)
grey	5	10	15	20	1.00	
blue	10	15	20	25	0.87	

Figure S2 (related to Figure 5). The Hodgkin-Huxley exponent '*m*' of NBQX/glutamate currents is influenced by the relative subconductance levels of states O1–O4.
NBQX/glutamate currents simulated using the scheme in **Figure 5A** (k_{NBQX} = 20 s^{-1}) demonstrating how the shape is influenced by subconductance levels of different relative magnitudes. Simulated curves were fitted (dashed lines) as in **Figure 5B**. Using measured subconductance levels for TARP-free AMPA/KARs (Rosenmund et al., 1998 - yellow) or those of Scheme 1 (Robert and Howe, 2003 - green) result in an *m* that approaches 2. Simulation using the sublevels from our single-channel recordings (red) yields a value of *m* (1.38) that is similar to that from our fits of TARPed macroscopic data (**Figure 5F**). When all states make a contribution proportional to their occupancy (grey) '*m*' is 1. If O1 makes a larger relative contribution (blue) '*m*' becomes less than 1 (for comparison, with GluA1/2(R)/γ-2, *m* = 0.91 ± 0.05).

FIGURE S3

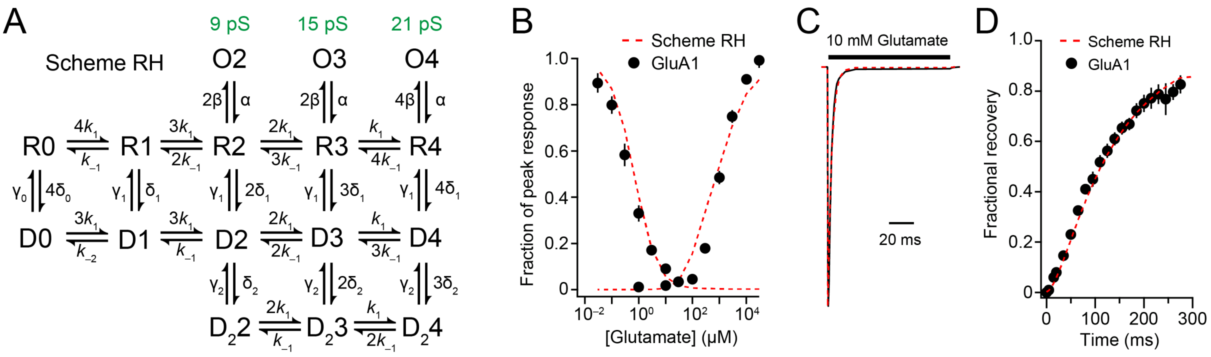

Figure S3 (related to Figure 6). A previously proposed model for GluA1 adequately describes our GluA1 data.
(**A**) Scheme RH, proposed by Robert and Howe (2003). All receptors (R0-R4) can desensitize (D0-D4). Receptors bound to two or more glutamate molecules (R2-R4) can additionally open (O) or enter a 'deep' desensitized state (D_2). With this scheme, a single set of rate constants can simultaneously approximate the two concentration response relationships (**B**), desensitization kinetics (**C**), and recovery kinetics of GluA1 (**D**) (red dashed lines). The following rate constants (constrained to be within 20% of previously published values) were used: $k_1 = 1.6 \times 10^7\ M^{-1}s^{-1}$; $k_{-1} = 7400\ s^{-1}$; $\alpha = 2600\ s^{-1}$; $\beta = 9600\ s^{-1}$; $\delta_1 = 1500\ s^{-1}$; $\gamma_1 = 9.1\ s^{-1}$; $\delta_2 = 170\ s^{-1}$; $\gamma_2 = 42\ s^{-1}$; $\delta_0 = 0.003\ s^{-1}$; $\gamma_0 = 0.83\ s^{-1}$.

FIGURE S4

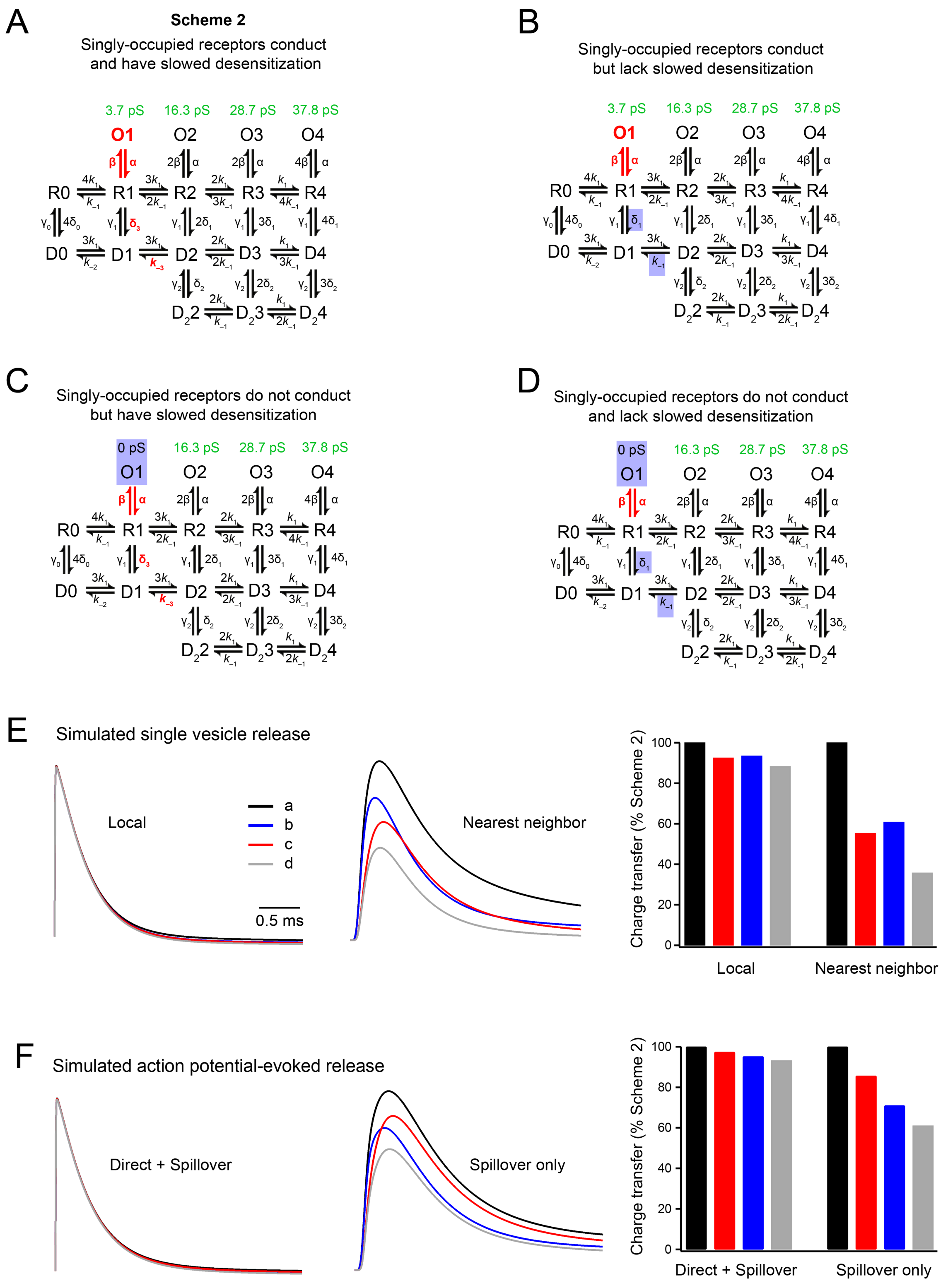

Figure S4 (related to Figure 7). Effects of γ-2 on both gating and desensitization of singly occupied receptors specifically increase charge transfer due to spillover.
(**A-D**) Kinetic schemes examining the relative contribution of changes to the conductance and kinetic behavior of singly occupied receptors (O1) to simulated synaptic charge transfer. (**A**) Scheme 2. (**B**) Scheme 2, altered to remove the slower desensitization of singly-liganded channels. (**C**) Scheme 2, altered to remove the

conductance of O1. (**D**) Scheme 2 with changes from A and B together. Blue boxes highlight the changed parameters. (**E**) Simulated single vesicle-induced currents resulting from kinetic schemes A-D (unboxed parameters as in **Figure 6**) using the glutamate waveforms shown in **Figure 7**. (**F**) As E, but for simulated action potential-evoked release. For both E and F, none of the scheme changes had marked effects on the charge transfer following local/direct activation (due to the high receptor occupancy in these conditions), but charge transfer of indirect activation was decreased by each of the individual changes and to a greater extent when both changes were made.

SUPPLEMENTAL EXPERIMENTAL PROCEDURES

Heterologous expression

We expressed recombinant AMPAR subunits and TARPs (plus EGFP) in HEK293 cells maintained under standard protocols, as described previously (Soto et al., 2007). AMPAR subunit cDNAs (rat) were 'flip' splice variants and the GluA2 forms were additionally R/G edited. Heteromeric AMPARs and AMPAR/TARP combinations were transfected at a cDNA ratio of 1:2 (GluA1:GluA2 and AMPAR subunit:TARP). The GluA1_γ-2 tandem consisted of full-length GluA1 and a 9 amino-acid linker (GGGGGEFAT) before the start codon of full-length γ-2. Transient transfection was performed using Lipofectamine 2000 (Life Technologies). Cells were split 12–24 h after transfection and plated on glass coverslips in the presence of 50 μM NBQX (Tocris-Abcam) to avoid AMPAR-mediated toxicity. Electrophysiological recordings were performed 18–24 h later.

Electrophysiology

Patch-clamp electrodes were pulled from borosilicate glass (1.5 mm o.d., 0.86 mm i.d.; Harvard Apparatus) and fire polished to a final resistance of 8–12 MΩ. For outside-out patches the 'external' solution contained 150 mM NaCl, 1 mM $CaCl_2$, 1 mM $MgCl_2$ and 10 mM HEPES, pH 7.4. The 'internal' solution contained 135 mM CsF, 33 mM CsOH, 11 mM EGTA, 10 mM HEPES, 2 mM $MgCl_2$ and 1 mM $CaCl_2$, pH 7.4 supplemented with 100 μM spermine tetrahydrochloride (Tocris Bioscience), except for NBQX/glutamate recordings where the internal solution contained 125 mM CsCl, 2.5 mM NaCl, 1 mM CsEGTA, 10 mM HEPES and 20 mM Na_2ATP. Recordings were made from outside-out patches at 22–25°C using an Axopatch 200A amplifier (Molecular Devices). Currents were recorded at –60 mV unless otherwise stated, low-pass filtered at 10 kHz and digitized at 20 kHz (Digidata 1440A interface with pClamp 10 software; Molecular Devices).

Rapid agonist application to excised patches

Rapid agonist application was achieved by switching between continuously flowing solutions, as described previously (Soto et al., 2014). Solution exchange was achieved by moving an application tool – made from either theta glass (2 mm outer diameter; Hilgenberg) or custom triple-barreled glass (Vitrocom) – mounted on a piezoelectric translator (Physik Instrumente) (MacLean et al., 2014; Robert and Howe, 2003). At the end of each experiment, the adequacy of the solution exchange was tested by destroying the patch and measuring the liquid-junction current at the open pipette (10–90% rise time typically 150–250 μs).

Data analysis

Concentration-response curves. Peak and steady-state concentration-response curves were constructed following application of multiple concentrations of glutamate to individual patches. Sensitivity to desensitization was derived from peak responses to 10 mM glutamate following equilibrium with various test concentrations of glutamate. Records were analyzed using Igor Pro 6.35 (Wavemetrics) with Neuromatic 2.8 (http://www.neuromatic.thinkrandom.com/), and curves from individual patches or pooled data were fit using the Hill equation to determine the concentration of glutamate generating the half-maximal response (EC_{50}) or half-maximal inhibition (IC_{50}). Each individual experiment was fitted separately and results were re-normalized to the peak of the fit. Pooled averages of the re-normalized results were refit for display only.

Conductance-voltage relationships. Responses to 2 s applications of 10 mM glutamate were obtained at various voltages between –110 mV and +60 mV. Peak and steady-state conductance-voltage (*G-V*) relationships were fitted with the Boltzmann equation:

$$G = G_{\mathrm{max}}\left(\frac{1}{1+\exp\left((V_{\mathrm{m}}-V_{½})/k\right)}\right),$$

where G_{max} is the conductance at a sufficiently hyperpolarized potential to produce full relief of polyamine block, V_m is the membrane potential, $V_{½}$ is the potential at which 50% of block occurs, and k is a slope factor describing the voltage dependence of block (the membrane potential shift necessary to cause an e-fold change in conductance). Each individual experiment was fitted separately and data were re-normalized to the peak of the fit. Pooled averages of the re-normalized results were then refit for display only.

Fluctuation analysis. To deduce channel properties from macroscopic responses, glutamate (10 mM) was applied to outside-out patches (100 ms duration, 1 Hz). Non-stationary fluctuation analysis was performed as previously described (Soto et al., 2007) but using only the first 95% of the current decay. The ensemble variance of all successive pairs of current responses was calculated and the single-channel current (i) and total number of

channels (N) were then determined by plotting this ensemble variance (σ^2) against mean current ($\bar{I}$) and fitting with a parabolic function:

$$\sigma^2 = i\bar{I} - \bar{I}^2/N + \sigma_B^2 ,$$

where σ_B^2 is the background variance (Sigworth, 1980). To estimate the conductance of channels open at steady-state we performed stationary fluctuation analysis by determining the mean current and variance of the steady-state current. A line was fit between the origin (current = 0, variance = σ_B^2) and the single data point for the steady-state current, the slope of which provided an estimate of the single-channel current (i). In both cases, the weighted-mean single-channel conductance was calculated from the single-channel current and the holding potential.

Entry into, and recovery from, desensitization. For high concentrations of glutamate desensitization was determined directly from the fit of the current. For low concentration of glutamate, the rate of peak current inhibition was assessed using a two-pulse protocol jumping from control, into the test concentration for varying times and finally to 10 mM glutamate. Recovery from steady-state desensitization was measured by jumping from the test glutamate concentration to 10 mM glutamate following various intervals in control solution. Entry into desensitization was fitted with a sum of two exponentials for all conditions. Recovery from desensitization data was pooled, normalized and for GluA1 was fitted using a monoexponential Hodgkin-Huxley type equation:

$$I_t = (1 - e^{\left(\frac{-t}{\tau}\right)})^m ,$$

where I_t is the peak current at a given interpulse interval (t), t is the recovery time constant, and m is an exponent the value of which corresponds to the number of kinetically equivalent rate-limiting transitions that contribute to the recovery time course. Fits were standardized with a time constant of 120 ms to more clearly assess the delay of recovery (m). For GluA1/γ-2 recovery, we first established that a lag was absent (Hodgkin-Huxley m = 0.99) then performed a global biexponential fit, according to:

$$I_t = A_f \exp(t/\tau_f) + A_s \exp(t/\tau_s) ,$$

where A_f and t_f are the amplitude and time constant of the fast component of recovery and A_s and t_s are the amplitude and time constant of the slow component. The recovery endpoint was constrained to 1 and fast and slow time constants were common to all conditions. Due to the low levels of desensitization, data acquired during recovery from 1 μM glutamate were excluded from the global fitting and were fitted independently, using the time constants derived from fits of the other three concentrations. The resultant weighted time constants were calculated as follows:

$$\tau_w = \tau_f \left(\frac{A_f}{A_f + A_s}\right) + \tau_s \left(\frac{A_s}{A_f + A_s}\right)$$

AMPAR activation following unbinding of NBQX. NBQX/glutamate currents were recorded by fast application of 10 mM glutamate following full block by 50 μM NBQX, all in the presence of 50 μM cyclothiazide. Macroscopic currents were binned by amplitude, yielding 100 data points calculated from the average time of all points in each bin. This approach gave equal weighting to each phase of the current for fitting. Data were fitted with a monoexponential Hodgkin-Huxley type function. Records suitable for single-channel measurement were filtered at 500 Hz. Stable sweeps were identified and conductances were assigned an occupancy (O1–O4) based on their position in the step-wise sequence. Cumulative all point histograms (0.025 pA binwidth) for each distinguishable conductance level per sweep were created in Igor Pro. Following normalization, histograms for each conductance state were averaged.

Kinetic Modeling

Kinetic simulations were performed in Scilab 5.5.0. (Scilab Enterprises; http://www.scilab.org) using the Q-matrix method (Colquhoun and Hawkes, 1982). For each set of rate constants, currents were calculated from the occupancies of all conducting states at given time points multiplied by their unitary current. GluA1/γ-2 NBQX/glutamate currents were fitted using the measured conductances of O1–O4 and by varying k_{NBQX} (**Figure 5e**). Least squares improvement of approximation of three concentration-response relationships, desensitization across all concentrations and recovery kinetics at 10 mM and 10 μM was performed within Scheme 2 (**Figure 6**). Normalized, averaged GluA1/γ-2 desensitization data was parsed (to 35 datapoints) to make the necessary computation manageable. The weighting of poorly fitted components was increased manually to facilitate convergence in subsequent rounds. For Scheme 2, k_{-2} and k_{-3} were constrained by microscopic reversibility.

SUPPLEMENTAL REFERENCES

Colquhoun, D., and Hawkes, A.G. (1982). On the stochastic properties of bursts of single ion channel openings and of clusters of bursts. Philos Trans R Soc Lond B Biol Sci *300*, 1-59.

Sigworth, F.J. (1980). The variance of sodium current fluctuations at the node of Ranvier. J Physiol *307*, 97-129.
